# Federated learning framework for medical image analysis with perspective-aware contrastive and mixture of experts

**DOI:** 10.3389/frai.2026.1807248

**Published:** 2026-06-11

**Authors:** Shradhanjali Das, K. Hemalatha

**Affiliations:** School of Computer Science and Engineering, Vellore Institute of Technology, Chennai, Tamil Nadu, India

**Keywords:** federated learning, medical image classification, perspective aware contrastive, mixture of experts, attention mechanism, attention contrastive model, multi perspective augmentation

## Abstract

Medical image analysis faces persistent challenges due to the distributed data, limited annotations, and variations in imaging modalities, acquisition protocols, and patient demographics. Centralized deep learning approaches compromise data privacy, while Federated Learning (FL) enables decentralized model training without sharing raw data. However, conventional FL frameworks struggle with non-IID distributions and heterogeneous clinical environments, limiting their generalization and stability. We propose FedPAC-ME, a novel Federated Learning Framework for Medical Image Analysis that integrates Perspective-Aware Contrastive Learning with a Mixture of Experts (MoE) architecture to address heterogeneity and data imbalance. The framework introduces Multi-Perspective Augmentation (MPA) to emulate diverse clinical views, and a Perspective-Aware contrastive Module (PACM) that aligns representations across modalities and clients. Additionally, a Mixture of Experts routing layer dynamically allocates specialized experts to client-specific data distributions, enhancing adaptability and collaboration across sites. A Perspective-Aware Contrastive Loss (PACL) further enforces cross-view consistency during local training while maintaining global coherence across institutions. Extensive experiments on the BraTS2020 multi-institutional brain tumor segmentation dataset demonstrate that FedPAC-ME achieves 98.80% accuracy, surpassing state-of-the-art FL baselines by over 2.5%. These results confirm the framework's effectiveness in improving feature alignment, generalization, and privacy preservation under diverse clinical conditions.

## 1 Introduction

Federated Learning has an important role in the modern healthcare system, with the rapid growth of distributed data generation across devices and institutions. It is a promising solution for training deep learning models while preserving privacy (Shahzad et al., 2024; Gosselin et al., 2022). Unlike traditional centralized techniques, FL allows multiple devices or institutions to train a shared model without sending their data to a central server, which is particularly valuable in privacy-sensitive domains such as healthcare, finance, and smart environments (Albalawi et al., 2024; Vepakomma et al., 2018). However, training high-performing models in such settings remains a challenge due to data heterogeneity, non-IID (non-independent and identically distributed) data distributions, and limited availability of labeled data across clients (Rieke et al., 2020; Joshi et al., 2022).

To help with these challenges, contrastive learning has recently become popular. It has shown remarkable representation learning improvements, especially in self-supervised and semi-supervised settings (Guo et al., 2023; Wu et al., 2024). By contrasting positive and negative pairs, models can learn discriminative features even with minimal supervision. However, integrating contrastive learning into federated settings is non-trivial, as it often leads to issues like representation misalignment across clients and lack of global consistency (Gosselin et al., 2022; Chen et al., 2024). These limitations highlight the need for novel strategies that can bridge local learning dynamics with global representation alignment in a privacy-preserving manner.

To address these limitations, we propose FedPAC-ME, a novel federated learning framework that tightly couples perspective-aware augmentation, client-specialized experts routing, and dual-stage contrastive learning. Rather than treating contrastive learning, the attention mechanism, and expert modeling as modular add-ons, FedPAC-ME integrates them into a unified design where multi-view consistency guides expert activation, and attention-weighted feature extraction is aligned across decentralized clients. This creates a mutually reinforcing loop that improves representation robustness, especially under the challenges of data heterogeneity and privacy constraints in medical imaging.

The proposed method, FedPAC-ME, begins at the client level, where an MHSA (Multi-Head Self-Attention) mechanism enables the model to focus on the most important parts of the data. A MoE module allows the model to handle different types of data, enabling effective feature extraction. Local contrastive learning is applied using a PACL that aligns representations from multiple augmented views of the data. After local training, client models share only their learned parameters with a central server, where Federated Averaging (FedAvg) is used to aggregate the updates. The global model then undergoes a second round of global contrastive learning, enhancing the alignment and consistency of shared representations across all clients.

This dual-stage contrastive learning, combined with perspective-aware augmentation and attention-based modeling, enables the framework to learn robust, generalized representations in a federated learning setting. The proposed approach effectively addresses the challenges of feature inconsistency, personalization, and limited supervision in decentralized environments.

**The following concisely outlines this work's main contributions:**

I. **Novel integration of multi-perspective contrastive learning and expert personalization:** We propose FedPAC-ME, the first federated learning framework to combine contrastive learning across augmented views with client-specific expert selection, enabling both global representation alignment and local adaptability under strict privacy constraints.
II. **Multi-perspective augmentation with adversarial, spatial, and perturbation views:** We introduce a tri-view augmentation strategy (MPA) combining perturbed (PCV), spatially shifted (SSV), and GAN-based adversarial views (GAV) to simulate real-world medical variability and enhance robustness against non-IID data distributions.
III. **Attention-driven expert routing for heterogeneous clients:** A Mixture of Experts module integrated with MHSA enables each client to adaptively route features through specialized expert paths based on semantic relevance, improving feature diversity and learning efficiency.
IV. **Dual-stage contrastive alignment for cross-client consistency:** We introduce a two-stage contrastive scheme for local alignment of augmented features at the client level and global representation alignment across institutions via Perspective-Aware Contrastive Loss (PACL), ensuring personalization and inter-client coherence without sharing sensitive data.

The rest of this paper is organized as follows. Section 2 provides a detailed review of related work and contemporary methods in FL, Contrastive Learning, and Attention Mechanism. Section 3 presents the proposed FL framework, including the FedPAC-ME methodology, MHSA and MoE, PACL, and global model aggregation strategy. Section 4 discusses the experimental results, including performance evaluation and comparisons with existing techniques. Finally, Section 5 concludes the paper and outlines potential directions for future research.

## 2 Related work

Recent advances in medical image analysis have been significantly shaped by the intersection of privacy-preserving deep learning models. FL, in particular, has emerged as a promising solution to address privacy concerns by enabling collaborative training across decentralized medical institutions without the need to share raw data. However, there are practical challenges in FL, such as data heterogeneity, label scarcity, limited annotations, and domain-specific variability. Various studies have been put forward to address these challenges. This section provides an overview of recent contributions to FL in medical imaging, with a focus on the roles of attention mechanisms and contrastive learning.

### 2.1 Review methodology

For literature selection, a **scoping review methodology** was utilized to systematically identify, screen, and curate relevant studies. This ensured comprehensive coverage of recent developments in privacy-preserving federated learning for medical image analysis, with particular emphasis on attention-based, contrastive, and semi-supervised approaches.

#### 2.1.1 Search strategy

A comprehensive literature search was conducted across multiple academic databases, including **IEEE Xplore, SpringerLink, ScienceDirect, PubMed, and Google Scholar**. The search was limited to publications from **2017 to 2025** to ensure coverage of recent developments in federated learning and medical imaging.

The following search keywords and combinations were used:

- “Federated Learning AND Medical Imaging”
- “Privacy-Preserving Deep Learning AND Healthcare”
- “Federated Learning AND Contrastive Learning”
- “Federated Learning AND Attention Mechanism”
- “Semi-Supervised Federated Learning AND Medical Image Segmentation”

#### 2.1.2 Inclusion and exclusion criteria

The collected studies were filtered based on the following criteria:

**Inclusion criteria:**

- Peer-reviewed journal articles and conference papers
- Studies focusing on federated learning in medical imaging
- Works addressing privacy-preserving techniques, contrastive learning, or attention mechanisms
- Publications in English within the selected time frame

**Exclusion criteria:**

- Non-peer-reviewed articles, abstracts, or incomplete studies
- Studies not related to medical imaging applications
- Papers lacking sufficient methodological details

#### 2.1.3 Study selection process

**Figure 1:**
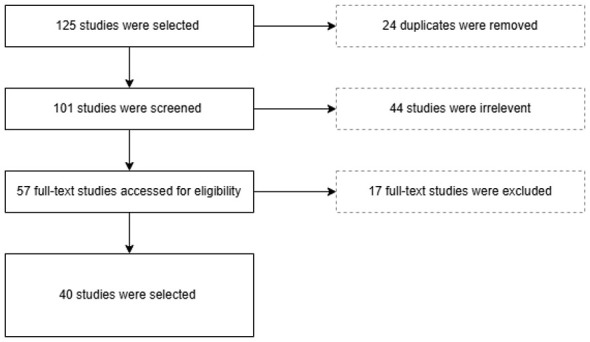
PRISMA framework.

The study selection process followed a structured screening approach inspired by the **Preferred Reporting Items for Systematic Reviews and Meta-Analyses (PRISMA)** framework show in Figure 1. Initially, relevant papers were identified through database searches. Duplicate entries were removed, followed by title and abstract screening. Full-text reviews were then conducted to ensure relevance and quality.

A total of approximately **125 papers** were initially identified, out of which **40 studies** were selected after applying the inclusion and exclusion criteria.

#### 2.1.4 Data extraction and categorization

For each selected study, key information such as methodology, dataset type, model architecture, privacy mechanisms, and performance metrics were extracted and analyzed.

The selected works were categorized into the following thematic groups:

- Federated Learning in Medical Imaging.
- Attention Mechanisms in Federated Settings.
- Contrastive Learning in Federated Settings.
- Semi-Supervised and Hybrid Federated Models.
- Privacy-Preserving Techniques.

This categorization enables a structured comparison of existing approaches and highlights the strengths and limitations of each research direction.

### 2.2 Federated learning in medical imaging

FL has emerged as a powerful platform for privacy-preserving medical image analysis by enabling collaborative model training without sharing sensitive patient data. It addresses data management and security challenges in multi-institutional collaboration. Joynab et al. (2024) showed how FL can be used to classify cervical cancer using Pap smear images. They got 94.36% accuracy when the data was evenly distributed (IID) and 78.4% when the data was uneven (non-IID), showing how different types of data can affect results. Similarly, Naga Srinivasu et al. (2024) used pre-trained CNN models in their FL setup, which helped improve accuracy in medical image classification, even when there wasn't much labeled data available.

Ahmed and Giordano (2022) developed a federated transfer learning method that reduces computational overhead needed while still keeping data private and results accurate, especially in resource-constrained settings. Cao et al. (2023) used a technique called homomorphic encryption, which strengthens privacy during training without reducing the model's accuracy. Ullah et al. (2024) created a flexible FL method for medical imaging that allows devices with on-and-off connections to still take part in smart healthcare systems. Yue et al. (2024) designed a special FL framework that can deal with class imbalance (when some classes have more data than others) by using dynamic feature fusion networks. Lei et al. introduced a federated learning model using Transformers to help diagnose Alzheimer's disease. Their method could successfully learn and transfer features from MRI scans taken at different hospitals (Lei et al., 2023). Yan et al. (2023) addressed the challenge of different types of data by creating a label-efficient, self-supervised FL method that works well with diverse medical images. Xu et al. (2023) worked on FL for segmenting multiple organs, even when the labels were inconsistent, showing that FL can adapt to tricky labeling situations.

In addition, Naga Srinivasu et al. (2024) enhanced medical image classification performance by incorporating pre-trained models into the FL pipeline. Their results suggest that transfer learning significantly boosts performance when labeled data is scarce, making it a valuable strategy for FL-based medical image analysis.

FL has also been used for more complex tasks, like combining different types of data and using graphs for classification. Lin et al. (2023) used Graph Convolutional Networks (GCNs) to better understand the relationships in the data, which helped improve results compared to traditional CNNs. These studies demonstrate that FL is a robust and secure approach for collaborative medical image analysis.

Despite these efforts, many FL approaches still struggle with highly non-IID data and label scarcity, especially in segmentation tasks. Moreover, most methods rely on global aggregation without sufficient local adaptation or personalization, which can lead to degraded performance in real-world deployments.

### 2.3 Attention mechanisms in federated settings

Adding attention mechanisms to FL helps the model focus on the most important parts of the data, which is useful when the data comes from different sources or varies a lot. Irfan et al. (2024) built a federated fusion model that uses attention to improve how clients learn together. Their approach helps the model focus on the most important features, making it more reliable and accurate when classifying medical images.

Shen et al. (2022) took this idea further by using Vision Transformers with a partly personalized attention system to classify chronic lung disease (COPD). Their model used shared layers for learning common patterns and separate parts for each client, which helped balance personal accuracy and overall learning, even when the data was very different across clients (non-IID).

Similarly, Bdair et al. (2024) used attention mechanisms in image-to-image translation to help with radiotherapy. Their spatial self-attention method helped the FL models focus on the important parts of the body, leading to better-quality images and improved treatment planning.

These approaches demonstrate the effectiveness of the attention mechanism in improving FL performance.

### 2.4 Contrastive learning in federated settings

Contrastive learning is becoming popular in FL because it helps models learn better features by distinguishing between similar and dissimilar images. This makes the model more general and effective across different clients. Liu et al. (2023) created FedCL, a contrastive FL method that utilizes contrastive loss to enhance global model alignment and convergence across multiple centers. The model learns to group similar images together and separate different ones, which improves its overall accuracy and ability to work well across multiple hospitals or institutions. Their approach also helps deal with problems like uneven data and class imbalance.

Yang et al. (2024) introduced a method called Dense Contrastive-based Federated Learning (DCFL) to better match global and local features for tasks like detecting lung nodules. Their approach improved recall by 6.07%, showing that contrastive learning can increase performance even when there is not much labeled data.

Self-supervised methods like SelfFed, created by Ali Khowaja et al. (2025), used a Swin Transformer-based encoder to combine contrastive learning with self-supervised federated learning. Their method effectively handles diverse and unlabeled medical data in federated learning. The transformer-based contrastive framework enabled learning from unlabeled medical images, improving representation quality and classification accuracy.

Similarly, Luo and Wu (2022) introduced FedSLD, which uses shared label information to guide training, helping the model to learn better with different types of data. These studies show that combining contrastive learning with FL can improve performance, especially when there are few labels and the data are very different between clients.

However, most existing federated contrastive learning frameworks like FedCL and DCFL assume relatively balanced distributions or rely on full-label supervision at the local level.Under non-IID or label-scarce settings, their performance deteriorates due to poor feature alignment and insufficient global consistency. SelfFed alleviates this to some extent through Swin Transformer backbones, yet lacks effective data augmentation diversity or personalization strategies. Our FedPAC-ME addresses these gaps by incorporating perspective-aware data augmentation, multi-head self-attention, and a Mixture of Experts to ensure robust feature learning across diverse and low-label environments.

### 2.5 Other models in federated learning

Due to the scarcity of labeled medical data, recent research has focused on integrating semi-supervised learning (SSL) with federated learning to effectively utilize both labeled and unlabeled data (Ali Khowaja et al., 2025; Wang P. et al., 2024). Wu et al. proposed a prototype-based pseudo-labeling approach combined with contrastive learning, demonstrating strong performance in medical image segmentation tasks, including COVID-19 datasets. This highlights the importance of pseudo-labeling in enhancing learning from limited annotations.

Building on this direction, several works aim to improve the quality and reliability of pseudo-labels. Qiu et al. (2023) introduced a denoising strategy to refine noisy pseudo-labels, leading to improved segmentation performance in federated semi-supervised settings. Similarly, Guo et al. (2023) proposed a dual class-aware contrastive framework that leverages confidence-based pseudo-labels along with both local and global class representations to guide learning more effectively. Wicaksana et al. (2023) further extended this idea through FedMix, which adaptively combines ground-truth labels with pseudo-labels to improve segmentation accuracy.

In addition to pseudo-label refinement, other approaches address data heterogeneity and personalization challenges in federated learning. (Wang J. et al. 2024) proposed FedDP, a dual-personalization framework designed to account for client-level variability while improving segmentation performance. Chen et al. (2024) incorporated attention mechanisms in vertical federated learning to effectively fuse metadata and image features, enhancing semi-supervised learning performance. Furthermore, Sun et al. (2024) introduced FKD-Med, which leverages knowledge distillation to improve both privacy and communication efficiency in federated medical image segmentation.

Beyond federated semi-supervised approaches, Multiple Instance Learning (MIL) has been explored as an alternative paradigm for handling weakly labeled medical data. MIL enables models to learn from bag-level annotations rather than precise pixel-level labels, making it particularly suitable for scenarios with limited detailed annotations. For example, MIL has been applied to diabetic retinopathy classification tasks (Vocaturo and Zumpano, 2021), while heuristic optimization strategies have been proposed to improve instance selection and classification performance (Fuduli et al., 2022). Additionally, AI-based diagnostic systems have demonstrated effectiveness in ultrasound-based breast cancer detection (Li et al., 2022), highlighting the broader applicability of learning techniques under weak supervision.

### 2.6 Privacy-preserving techniques in federated learning

Protecting data privacy is a key concern in federated learning for medical imaging. Mantey et al. (2024) investigated the use of homomorphic encryption to secure the training process in medical recommender systems, demonstrating that encrypted computation is practical for handling sensitive healthcare data. Likewise, Liu et al. (2024) proposed a detailed privacy-focused framework that includes secure authentication methods, offering strong protection for federated learning in Internet of Medical Things (IoMT) settings.

Alongside encryption methods, Wang et al. (2023) suggested a privacy-friendly split learning model with differential privacy, designed for large-scale image training and also useful for medical imaging. Mu et al. (2024) developed an explainable federated learning approach using causal learning and blockchain, which improves both data privacy and the ability to understand model decisions.

### 2.7 Time complexity and parameter overhead in federated settings

Most classical FL algorithms, such as FedAvg and FedProx, maintain low computational overhead because they rely on simple local SGD updates and global model averaging. Their per-round time complexity is dominated by local training, typically *O*(*E*.*N*.*d*), where *E* is the number of local epochs, *N* is the number of samples per client, and *d* is the number of model parameters. The communication overhead for each round is also minimal, requiring only the transmission of the full model parameters *O*(*d*) between clients and server.

However, recent FL research in medical imaging has highlighted that while FedAvg/FedProx are computationally lightweight, they often struggle with complex tasks such as lesion classification or segmentation under non-IID distributions. For example, Umirzakova et al. (2025) demonstrated the need for more expressive deep learning architectures when working with MRI-based multiple sclerosis lesion classification, reinforcing that simple aggregation strategies may fall short in capturing fine-grained medical features.

In comparison, advanced frameworks, such as those incorporating attention mechanisms, contrastive learning, or mixture-of-experts modules, introduce moderate additional complexity. These models typically increase parameter counts through multi-head attention blocks or expert networks, resulting in higher *d* and thus larger communication payloads. Despite this, they provide substantial gains in representation quality, robustness to heterogeneity, and performance under label-scarce or non-IID conditions. Thus, while classical methods like FedAvg and FedProx offer efficiency, more sophisticated architectures strike a better balance between computational cost and accuracy in practical medical imaging scenarios.

### 2.8 Research gaps and contributions

In previous studies, federated learning has been applied with contrastive learning techniques; however, challenges remain in effectively learning meaningful representations from non-IID data, achieving consistency across clients, and handling limited labeled data in a privacy-preserving manner. Most existing methods lack mechanisms to align representations both locally and globally, and often do not consider the varying perspectives within augmented views of the data.

The proposed framework addresses these limitations through:

- This framework introduces a dual-level contrastive learning approach that ensures feature consistency throughout the process. Applying contrastive training at both levels helps the model learn robust representations that generalize well across various clinical sites and imaging modalities, including CT, MRI, and X-rays. This is particularly useful for medical imaging tasks, where differences in scanner type, imaging protocols, and patient population can cause domain shifts.
- By using numerous augmented views of the same medical image (e.g., rotated, contrast-enhanced, or cropped views), the proposed PACL improves representation learning. Even with a small amount of labeled data, it guarantees that semantically related medical patterns (such as tumors, lesions etc) are closely mapped in the feature space, improving lesion border detection and semantic segmentation performance.
- Highly detailed, spatially complex formations are frequently seen in medical images. Each client has an attention-driven architecture that uses MoE and MHSA modules to address this. This improves the detection and segmentation of small or ambiguous regions, such as early-stage cancers or overlapping tissues, by enabling the model to adaptively focus on important anatomical traits and route image patches to specialized experts.
- A multi-perspective data augmentation strategy is applied to simulate diverse imaging conditions and anatomical variations. This includes augmentations like intensity scaling (to mimic different scanners), geometric transforms (to simulate anatomical variance), and synthetic noise (to reflect real-world artifacts). These augmentations enhance the model's robustness and generalization to unseen clinical environments, reducing overfitting and improving diagnostic reliability.

## 3 Methodology

**Figure 2:**
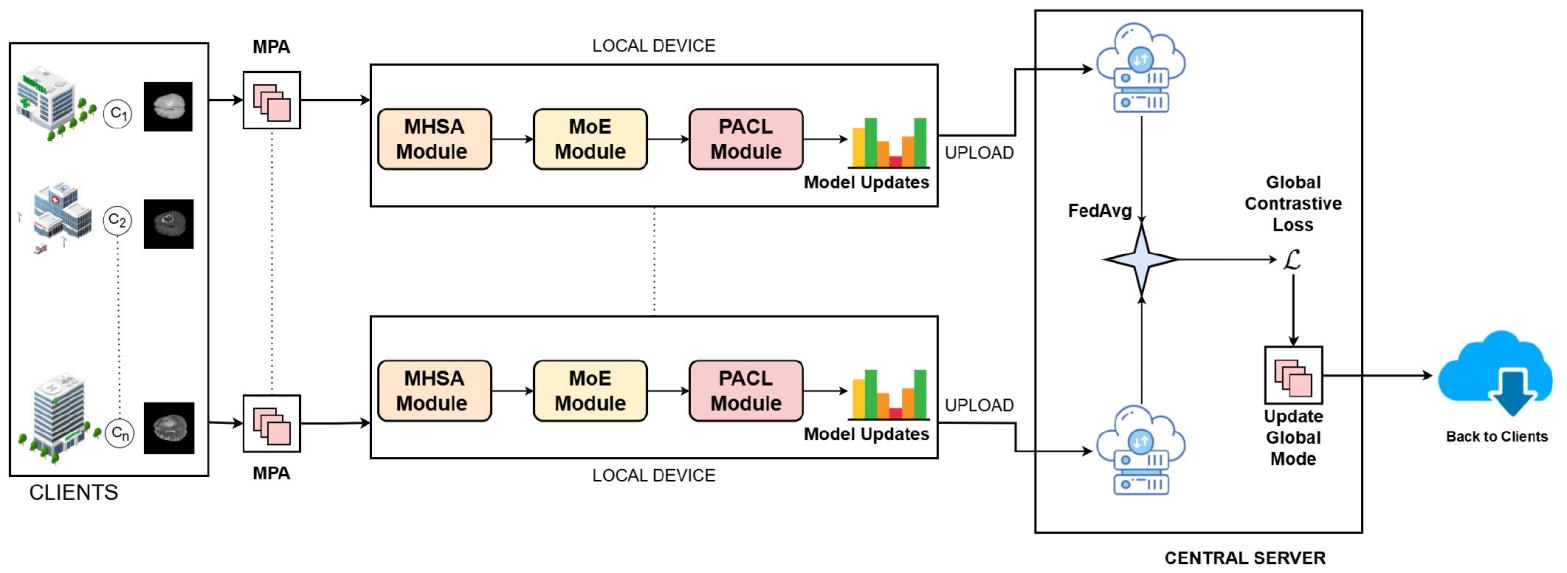
Overview of the proposed federated learning framework. Each client receives local medical image data (2D slices) and processes them through a sequence of modules, including MHSA for feature extraction, MoE for adaptive feature refinement, and PACL for contrastive representation learning. The clients compute local model updates, which are uploaded to the central server. At the server side, the received updates are aggregated using federated averaging, and a global contrastive loss is applied to align representations across clients. The updated global model is then redistributed back to all clients for subsequent training rounds.

We propose FedPAC-ME, a novel federated learning framework that uniquely integrates multi-perspective contrastive learning, a Mixture of Experts (MoE) routing layer, and self-attention to address challenges of heterogeneity and label imbalance in medical image analysis, as illustrated in Figure 2. Unlike existing FL frameworks that apply basic augmentation and pooling, our method explicitly models view diversity and specialization at both local and global levels through three key innovations: Multi-Perspective Augmentation (MPA) for view-rich contrastive learning, MoE-based expert selection per sample to deal with heterogeneous client distributions, and Global contrastive fine-tuning to improve generalization across clients. As illustrated in Figure 1, the data flow begins at the client side, where local medical images are transformed into feature representations and model updates. These updates are transmitted to the central server, where aggregation and global contrastive optimization are performed. The resulting global model is then shared back with all clients, enabling iterative federated training across distributed data sources.

The framework starts with local training at each client using multi-perspective data augmentation to generate diverse views of the same medical images. A combination of MHSA and MoE is applied to help the model focus on essential features and adapt to variations in the data. Then, PACL is used to improve the model's ability to distinguish between similar and different image representations. After local training, model updates are shared with the central server and combined using FedAvg. The aggregated global model then undergoes global contrastive learning to further align feature representations between clients. This process improves overall classification or segmentation performance while preserving data privacy, making the model effective across diverse and distributed medical datasets.

### 3.1 Code availability

The custom code developed for implementing the proposed FedPAC-ME framework is available in a public GitHub repository at: https://github.com/Shradha1023/FedPAC-ME.

A permanent, citable version of the code has been archived on Zenodo and assigned a DOI: 10.5281/zenodo.17883548, in accordance with Scientific Reports editorial policies. The archived version corresponds to the exact code used in this study's experiments. The code is released under the *MIT* license. No restrictions apply to accessing or using the code. Any dependencies and environment configurations are documented in the repository README.md.

### 3.2 Dataset description

The BraTS2020 challenge dataset (Bakas et al., 2017, 2018; Menze et al., 2015, 2020; Kaggle, 2020) comprises of multimodal MRI scans. All scans are provided in NIfTI formay (.nii.gz) and include the following four imaging modalities: T1-weighted (T1.nii) for structural details, contrast-enhanced T1 (T1ce.nii) to highlight active tumor regions, T2-weighted (T2.nii) for fluid-containing areas, and FLAIR (flair.nii) for detecting tumor-associated edema. The dataset also provides segmentation masks (seg.nii) that show the exact tumor areas, helping in model training and evaluation. It divides the tumor into different sub-regions: necrotic and non-enhancing tumor core, peritumoral edema, and GD-enhancing tumor. The dataset is organized into training and validation sets, with the training folder containing 369 patient samples. These multiple image types help in accurately detecting tumors, making the dataset useful for building and testing FL models while keeping patient data private.

To further evaluate the generalizability of the proposed framework, we incorporate an additional dataset consisting of gastric cancer histopathology tissue images (Lou et al., 2024). Unlike BraTS2020, which contains MRI-based brain tumor images, this dataset represents a fundamentally different imaging modality characterized by high-resolution microscopic texture, staining variability, and cellular-level structures.

The inclusion of this dataset introduces significant domain diversity, enabling evaluation under heterogeneous feature distributions and strengthening the assessment of model robustness in cross-modality federated learning scenarios.

### 3.3 Data preparation

Before training the model, the dataset is prepared by organizing it into client-specific folders, processing the images, and creating dataloaders for efficient training and validation. The dataset was distributed among multiple clients, *C* = {*C*_1_, *C*_2_, …, *C*_*n*_}, simulating a FL environment for decentralized medical imaging. Each client received a subset of the BraTS2020 dataset, ensuring a balanced distribution of patient scans.

Let $D_{i}={\left\{\left(x_{j},y_{j}\right)\right\}}_{j=1}^{m}$ denote the dataset assigned to client *C*_*i*_, where *x*_*j*_ represents an input MRI scan and *y*_*j*_ is the corresponding segmentation label. Each client's dataset contains patient-specific folders with multimodal MRI scans (FLAIR, T1ce, T2, T1, and segmentation masks). To simulate realistic federated learning conditions, we adopt a Dirichlet distribution-based non-IID partitioning strategy. Let K denote the number of classes and N the number of clients. For each class, data samples are distributed across clients according to a Dirichlet distribution parameterized by a concentration parameter α. The dataset was divided across 10 clients, *C*_*i*_:


$$\begin{array}{l} D=\overset{10}{\underset{i=1}{⋃}}D_{i} \end{array}$$ (1)

Where |*D*_1_|≠|*D*_2_|≠⋯≠|*D*_10_|

A validation set *D*_*val*_ was created from the BraTS2020 validation dataset to evaluate model performance.

Each MRI scan underwent pre-processing to standardize input data across clients. Given an image volume *X*∈ℝ^*H*×*W*×*D*^, the pre-processing steps include: Rescaling, where images were resized to (128x128) for computational efficiency, Normalization where each voxel intensity was normalized using mean μ = 0.5 and standard deviation σ = 0.5: *X*′ = (*X*−μ)/σ and Segmentation Mask Handling where missing segmentation masks were replaced with an empty mask $M_{0}\in {\mathbb{R}}^{H\times W\times D}$, ensuring uniformity in training.

A dataset class *D* was defined to load multimodal MRI scans, extract the middle axial slice, and stack them into a multi-channel representation:


$$\begin{array}{l} I_{i}=\left[X_{\text{FLAIR}},X_{\text{T1ce}}\right]\in {\mathbb{R}}^{2\times 128\times 128} \end{array}$$ (2)

Where, *X*_FLAIR_ and *X*_T1ce_ are the FLAIR and T1ce slices, respectively. The segmentation mask *Y*_*i*_ was binarized as:

*Y*_*i*_ = 1(*M*>0) where 1(.) is an indication function.

Each client's dataset was loaded using PyTorch Dataloaders, defined as:

*D*_*i*_ = *Dataloader*(*D*_*i*_, *batchsize* = 4, *shuffle* = *True*)

Similarly, a validation Dataloader was created for centralized evaluation:

*D*_*val*_ = *Dataloader*(*D*_*val*_, *batchsize* = 4, *shuffle* = *False*)

Structuring the dataset in this manner enables privacy-preserving federated learning, allowing each client to train on local data while contributing to a globally aggregated model without sharing raw patient scans.

### 3.4 Module integration and data flow

**Figure 3:**
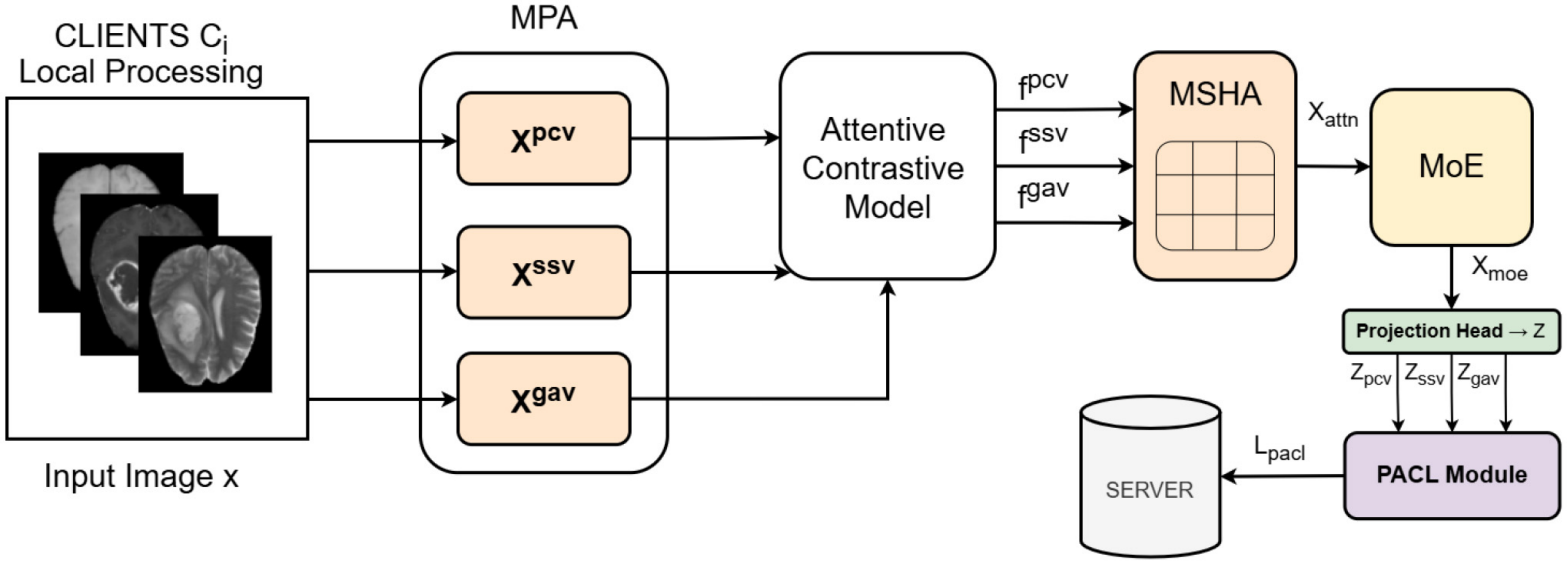
Integrated data flow of the FedPAC-ME framework, showing how modules interact from data input to global aggregation and contrastive alignment.

This section illustrates how the components are integrated within the proposed FedPAC-ME pipeline to form an end-to-end federated learning framework. The overall data flow from input MRI scans to global model aggregation is shown conceptually in Figure 3.

**Step 1: Input and Multi-Perspective Augmentation (MPA)**. Each client *C*_*i*_ begins with its local dataset *D*_*i*_ = {(*x*_*j*_, *y*_*j*_)}, where *x*_*j*_ represents an MRI slice. The image *x*_*j*_ is first passed through the Multi-Perspective Augmentation (MPA) module to create three distinct but semantically consistent augmented versions:


$$x^{pcv},x^{ssv},x^{gav}=\text{MPA}\left(x\right)$$

These augmentations, Perturbed, Spatially Shifted, and Gradient-based Adversarial Views, expand the representation space and simulate real-world medical variations such as noise, misalignment, and adversarial perturbations. All generated views, along with the original image, are then forwarded to the feature extraction stage.

**Step 2: Feature Extraction via Attention Contrastive Model (ACM)**. Each augmented view is processed by the Attention Contrastive Model (ACM), which consists of a DenseNet-121 backbone followed by flattening and global average pooling. This yields the base feature representations:


$$\begin{array}{r} f^{pcv},f^{ssv},f^{gav}=\text{DenseNet-121}\left(x^{pcv}\right),\text{DenseNet-121}\left(x^{ssv}\right),\\ \text{DenseNet-121}\left(x^{gav}\right) \end{array}$$

The output feature vectors are passed into the Multi-Head Self-Attention (MHSA) layer for spatial and contextual refinement.

**Step 3: Contextual Refinement using Multi-Head Self-Attention (MHSA)**. The MHSA module enhances the extracted features by modeling dependencies between spatial regions of MRI scans. Each view's flattened feature vector *X*_*flat*_ is transformed into attention-refined embeddings:


$$X_{attn}=LayerNorm\left(X_{flat}+MHSA\left(X_{flat}\right)\right)$$

This step enables the model to capture both local and global structural information, crucial for tumor boundary delineation and tissue differentiation.

**Step 4: Dynamic Routing through Mixture of Experts (MoE)**.

The attention-refined embeddings *X*_*attn*_ are input to the Mixture of Experts module, where a gating network determines which expert subnetworks are most suitable for the given input distribution. Using Top-*K* selection, the model adaptively routes each sample:


$$X_{moe}=\underset{i\in \tau }{\sum }g_{i}\cdot E_{i}\left(X_{attn}\right)$$

This selective routing mitigates data heterogeneity by allowing experts to specialize in modality-specific or institution-specific patterns while maintaining a shared representation space.

**Step 5: Embedding Projection and Perspective-Aware Contrastive Learning (PACL)**. The output of the MoE, *X*_*moe*_, is projected into a lower-dimensional embedding space *Z* through a projection head:


$$Z=\text{ProjectionHead}\left(X_{moe}\right)$$

For each original image and its three augmented views, PACL enforces representation consistency by minimizing the distance between embeddings of the same sample under different perspectives:


$$L_{\text{pacl}}=\frac{1}{\tau }\underset{v\in \left\{pcv,ssv,gav\right\}}{\sum }\left(1-\text{sim}\left(z,z_{v}\right)\right)$$

This ensures robustness to perturbations and enhances intra-class cohesion while maintaining inter-class separability.

**Step 6: Local Model Optimization**. Each client performs local optimization by minimizing a combination of the PACL loss and segmentation loss. The updated model parameters θ_*i*_ are then transmitted to the central server:


$$\theta_{i}^{t+1}=\text{LocalUpdate}\left(\theta_{i}^{t},L_{\text{pacl}},D_{i}\right)$$

**Step 7: Global Model Aggregation (FedAvg)**. Upon receiving all local updates, the server aggregates model weights using the Federated Averaging (FedAvg) algorithm:


$$\theta_{G}^{t+1}=\frac{1}{N}\overset{N}{\underset{i=1}{\sum }}\theta_{i}^{t+1}$$

This step integrates client knowledge while maintaining data privacy, forming an updated global model $\theta_{G}^{t+1}$.

**Step 8: Global Contrastive Alignment**. To further harmonize feature spaces across institutions, the aggregated model undergoes global contrastive fine-tuning. The global contrastive loss aligns embeddings across clients using positive and hard negative samples:


$$L_{\text{contrastive}}=-\frac{1}{m}\overset{m}{\underset{i=1}{\sum }}\log\frac{\exp\left(\text{sim}\left(z_{i},z_{j}\right)/\tau \right)}{\sum_{k=1}^{m}\exp\left(\text{sim}\left(z_{i},z_{k}\right)/\tau \right)}$$

This post-aggregation refinement ensures consistency in the embedding distribution across heterogeneous data sources.

**Step 9: Iterative Federation**. Finally, the refined global model is redistributed to all clients for the next training round. Each client continues local training using the updated parameters, enabling iterative improvement until convergence.

Through this tightly integrated pipeline, FedPAC-ME transforms raw MRI inputs into robust global representations via sequential interaction between MPA, ACM, MHSA, MoE, PACL, and FedAvg modules. The integration of local specialization and global alignment ensures both privacy preservation and cross-institutional generalization.

### 3.5 Multi-Perspective Augmentation (MPA)

The first step in our pipeline is Multi-Perspective Augmentation (MPA), which enhances the model's ability to generalize by applying several transformations to the input MRI image. While prior contrastive learning methods, such as SimCLR and MoCo, generate augmented pairs using random transformations, they do not tailor these augmentations to the specific needs of the medical imaging domain or the constraints of federated learning. MPA is a targeted augmentation module that creates three task-specific contrastive views, PCV, SSV, and GAV, that simulate realistic variations in MRI scans (noise, shifts, and adversarial attacks).

**Figure 4:**
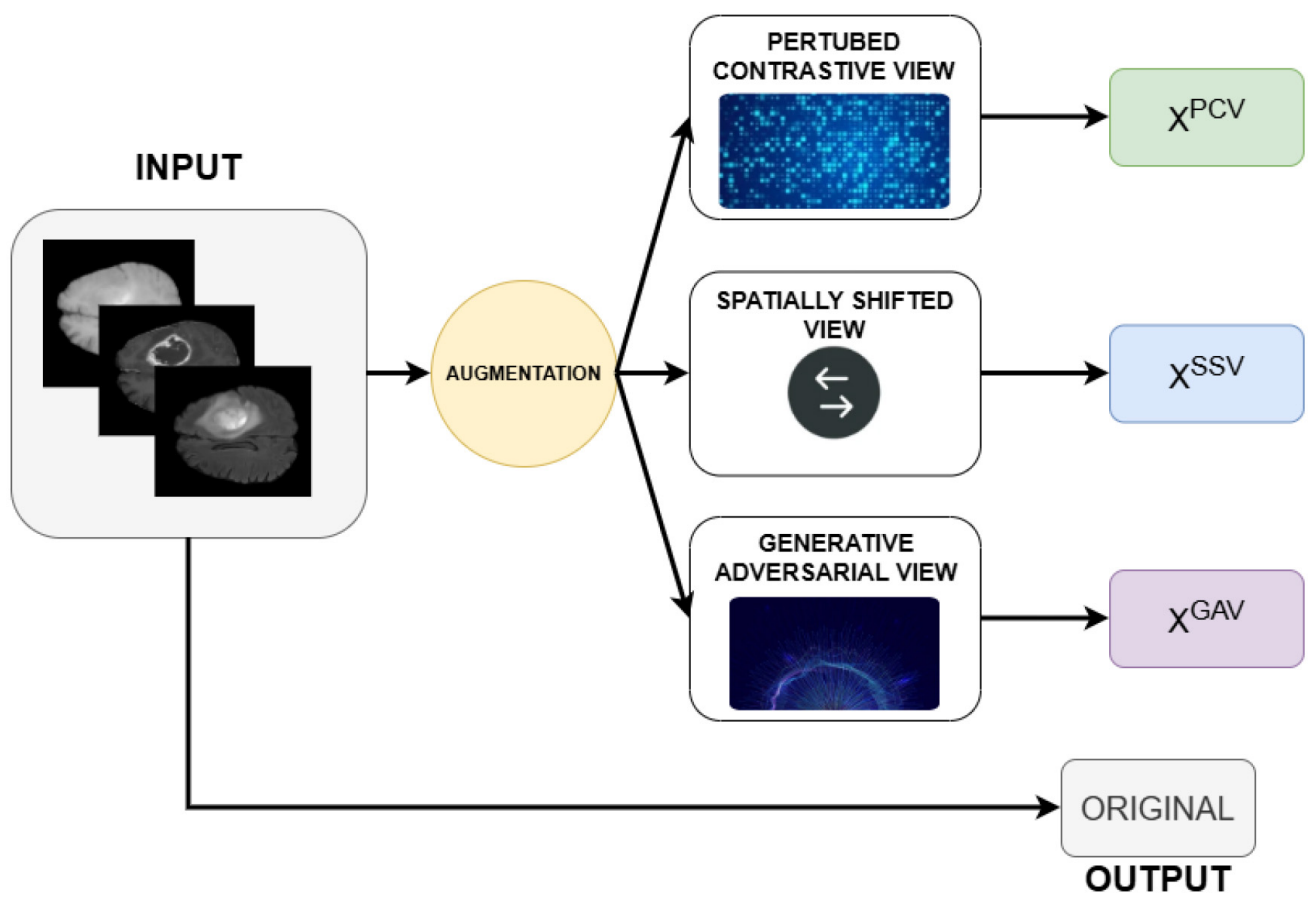
Multi-perspective augmentation architecture.

Figure 4 illustrates the architecture of the proposed PACL. First, the input medical image goes through different types of changes (augmentations) to create three new versions: Perturbed Contrastive View (PCV), which adds small noise to the image, Spatially Shifted View (SSV), which moves the image slightly in different directions, and Generative Adversarial View (GAV) crafted using adversarial perturbations to highlight model vulnerabilities. These three views give different perspectives of the same image. The original image is then compared with each of these views using cosine similarity to measure the similarity of their features.

MPA comprises three augmentation strategies designed to introduce the perspective of each input sample. Perturbed Contrastive View (PCV) simulates minor noise and intensity changes, Spatially Shifted View (SSV) enforces spatial invariance via pixel translation, and Gradient-based Adversarial View (GAV) encourages robustness against adversarial shifts. These views are passed through a shared attention-driven feature extractor followed by a Mixture of Experts (MoE) routing layer, and then projected into a common embedding space for contrastive learning.

Let the input image be *x*∈ℝ^*C*×*H*×*W*^.

1. **Perturbed Contrastive View (PCV)**

Random noise is added to the image to simulate small intensity variations. This augmentation is designed to help the model learn invariant features that are robust to slight intensity shifts in the image.


$$\begin{array}{l} x^{\text{pcv}}=x+\delta ,\;\delta \sim N\left(0,\sigma^{2}\right) \end{array}$$ (3)

2. **Spatially Shifted View (SSV)**

This augmentation randomly shifts the image by a small number of pixels in either the horizontal or vertical direction. This helps simulate small misalignments in the image that may occur due to patient movement or acquisition artifacts.


$$\begin{array}{l} x^{\text{ssv}}=\text{Roll}\left(x,\Delta x,\Delta y\right),\;\Delta x,\Delta y\in \left[-s,s\right] \end{array}$$ (4)

3. **Gradient-Based Adversarial View (GAV)**

The adversarial view is generated by applying the Fast Gradient Sign Method (FGSM) to the image. The FGSM generates perturbations in the image that maximize the model's prediction error, thus creating a challenging scenario for the model to learn from.


$$\begin{array}{l} x^{\text{gav}}=x+\epsilon \cdot \text{sign}\left(\nabla_{x}∥f\left(x\right){∥}_{2}\right) \end{array}$$ (5)

where f(x) is the intermediate feature representation and ϵ controls the perturbation magnitude.

The three augmented views are generated by the MPA module and then passed through a shared attention-driven feature extractor, followed by a Mixture of Experts (MoE) routing layer. This step ensures that the generated views are projected into a common embedding space for contrastive learning. The final output of the MPA is:


$$\begin{array}{l} x^{\text{pcv}},x^{\text{ssv}},x^{\text{gav}}=\text{MPA}\left(x\right) \end{array}$$ (6)

### 3.6 Attention Contrastive Model (ACM)

The next step in the pipeline is the Attention Contrastive Model (ACM). After augmentation, the generated views are passed into the ACM, which serves to extract deep features from the MRI images, refine those features using self-attention, and prepare them for subsequent processing. The core function of the ACM class is to generate robust feature representations that can later be used for contrastive learning.

**Step 1: Feature Extraction using DenseNet-121:** DenseNet-121 is a well-established architecture for extracting hierarchical features from images. In this step, the original and augmented images are processed by the DenseNet-121 network, which consists of multiple dense blocks. DenseNet-121 encourages feature reuse and ensures that each layer has access to all previous layer outputs.

Given the image *x*, the DenseNet model computes the feature map *f*(*x*), which is a high-dimensional tensor:


$$\begin{array}{l} f\left(x\right)=DenseNet-121\left(x\right) \end{array}$$ (7)

**Step 2: Flattening and Pooling:** After the DenseNet layers process the image, the output feature map is subjected to global average pooling, which converts the 2D feature map into a 1D feature vector. This vector captures the essence of the image while reducing its dimensionality:


$$\begin{array}{l} X_{flat}=GlobalAvgPool\left(f\left(x\right)\right)\in {\mathbb{R}}^{B\times 1024} \end{array}$$ (8)

Where *X*_*flat*_ is the flattened feature representation for each image in the batch. This 1D vector is the input to the next step in the pipeline, Multi-Head Self-Attention (MHSA).

**Step 3: Passing to Multi-Head Self-Attention:** The output of DenseNet-121 is then passed to the Multi-Head Self-Attention mechanism, which refines the features by allowing the model to focus on different parts of the input. Self-attention is a mechanism where each feature in the input is weighted by its relevance to other features, allowing the model to capture long-range dependencies and interactions between features.


$$\begin{array}{l} X_{attn}=MHSA\left(X_{flat}\right) \end{array}$$ (9)

Where $X_{attn}\in {\mathbb{R}}^{B\times 1024}$ is the attention-refined feature vector.

### 3.7 Multi-head self-attention

MHSA is a key part of the Transformer architecture, and it helps the model focus on different parts of the input at the same time. In medical image analysis tasks like brain tumor detection using the BRATS2020 dataset, MHSA is useful because it allows the model to understand both the small details and the overall structure of the image.

**Figure 5:**
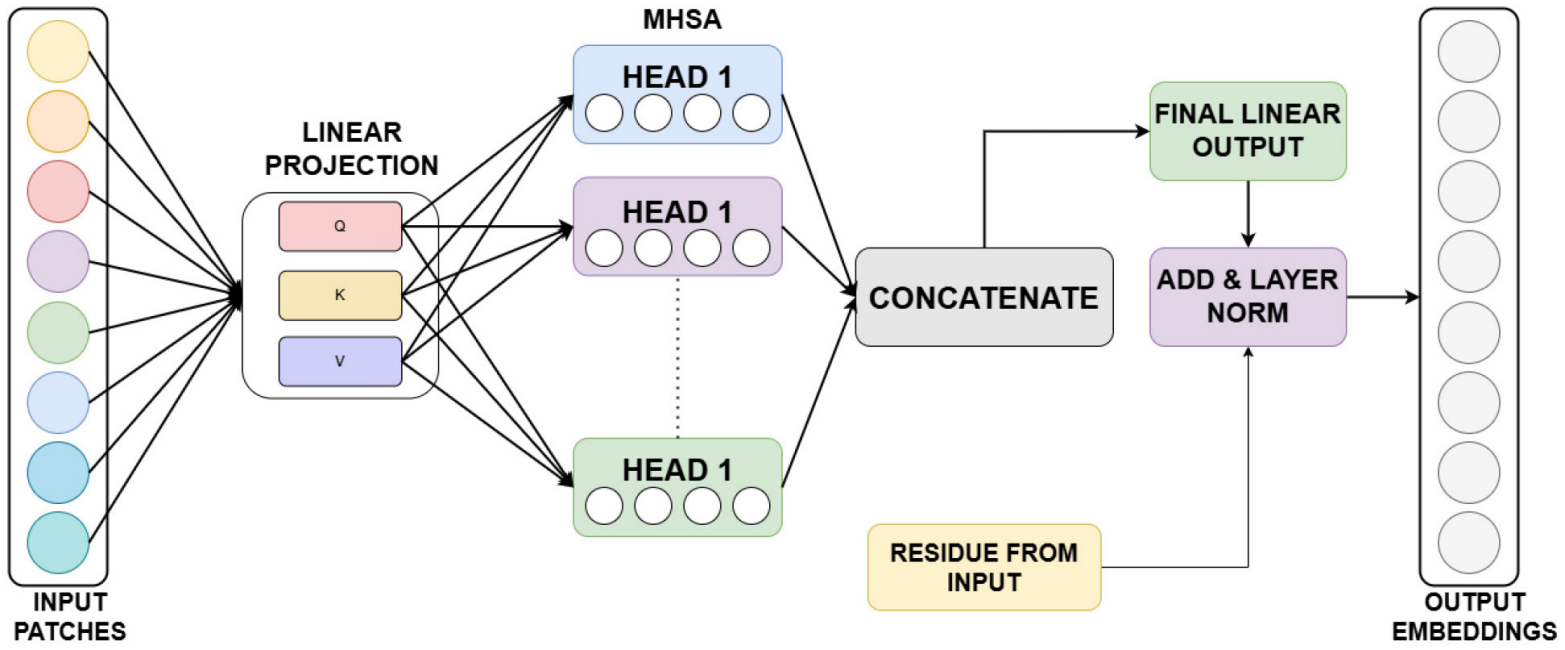
Multi-head self-attention architecture.

Figure 5 shows how the MHSA layer functions within the local model of each client in the FL setup. The figure starts with input patches, which are small sections of MRI images. These patches are shown as colored circles to represent labeled data. Each patch is first turned into three parts: Query (Q), Key (K), and Value (V) using a linear projection. These parts are then sent to different attention heads (head 1, head 1, up to head N). Each head learns to focus on different features of the images, helping the model understand the important areas better. These results from all heads are then combined. This combined output goes through a final linear layer, and then a residual connection adds the original input back to keep important information. Finally, a layer normalization step is applied to make training more stable. The result is shown as output embeddings, which are the final learned features.

Given an input feature representation *X*∈ℝ^*B*×*d*^, where *B* is the batch size and *d* is the embedding dimension, the MHSA mechanism first projects the input into query (*Q*), key (*K*), and value (*V*) matrices using learnable linear transformations:


$$\begin{array}{l} Q=XW_{q},K=XW_{k},V=XW_{v} \end{array}$$ (10)

Where $W_{q},W_{w}\in {\mathbb{R}}^{d\times d/8}$ and $W_{v}\in {\mathbb{R}}^{d\times d}$ are learnable projection matrices. Query (*Q*) represents the target information being sought in the image, Key (*K*) contains information about different features in the image, and Value (*V*) stores the actual feature values.

The model calculates an attention score based on the similarity between *Q* and *K*. This score determines which features are important. The attention scores are computed using the scaled dot-product mechanism:


$$\begin{array}{l} A\left(Q,K,V\right)=\text{Softmax}\left(\frac{QK^{T}}{\sqrt{d_{k}}}\right)V \end{array}$$ (11)

Where *d*_*k*_ = *d*/*h* is the key dimension. *h* is the number of heads. The outputs from all heads are concatenated and passed through a final linear layer:


$$\begin{array}{l} MHSA\left(X_{flat}\right)=Concat\left(head_{1},\ldots ,head_{h}\right)W^{o} \end{array}$$ (12)

Where, *head*_*i*_ = *A*(*Q*_*i*_, *K*_*i*_, *V*_*i*_) and *W*^*o*^∈ℝ^*d*×*d*^

Finally, a residual connection and layer normalization are applied to stabilize training:


$$\begin{array}{l} X_{attn}=LayerNorm\left(X_{flat}+MHSA\left(X_{flat}\right)\right) \end{array}$$ (13)

In contrast to standard CNN encoders, our ACM integrates multi-head self-attention with specialized experts (MoE) to handle spatial variability and data heterogeneity inherent in MRI scans from different institutions. This dual mechanism allows the model to focus on informative regions while dynamically routing samples to the most relevant expert subnetworks, enhancing robustness in the federated setup.

### 3.8 Mixture of experts

An MoE mechanism was integrated into the local model of each client to enhance learning across heterogeneous and imbalanced data distributions, particularly in medical image analysis. MoE helps the model specialize by activating different “expert” sub-networks for different inputs, allowing for better generalization and efficient learning in federated environments.

In federated environments, data distributions across clients are often non-IID and imbalanced, which makes shared models less effective. Inspired by routing networks and expert mixture models, we introduce a lightweight MoE module at each client, enabling local specialization without increasing global parameter size. Unlike prior FL works that use uniform models per client, our design activates only a subset of experts per input, allowing the model to adaptively specialize to local data without sacrificing generalization.

**Figure 6:**
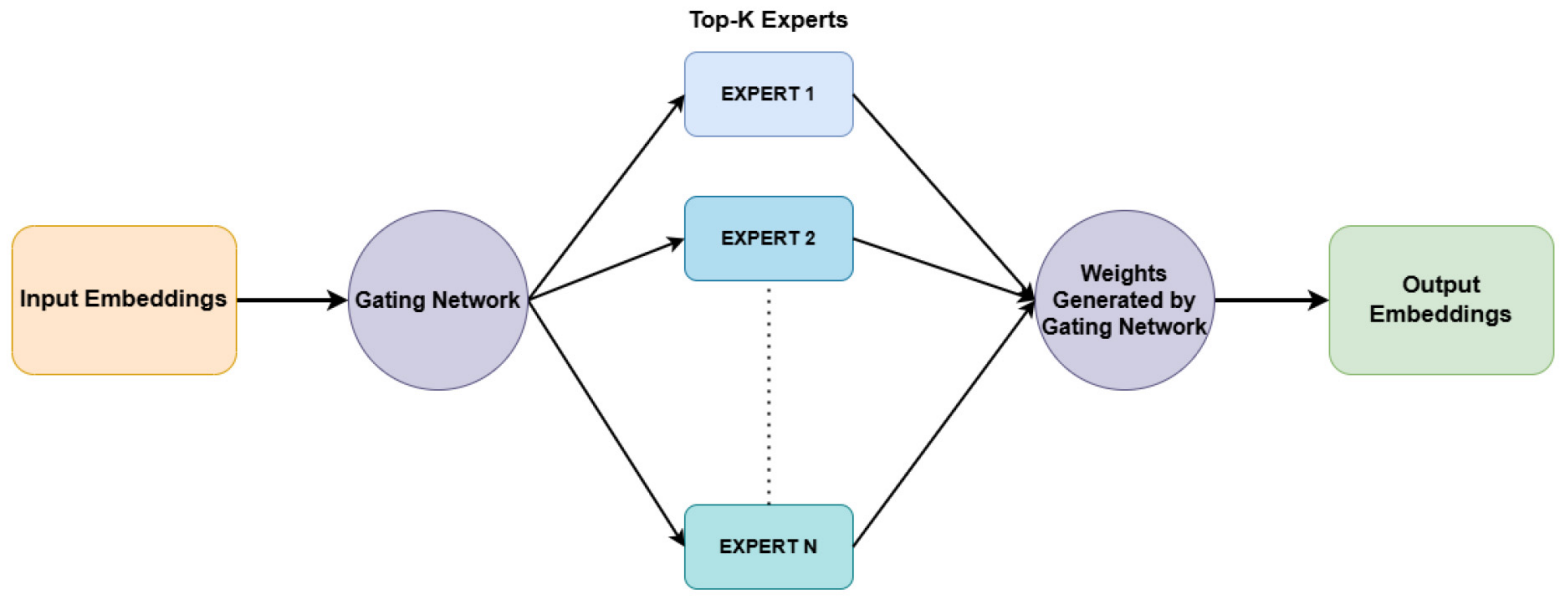
Mixture of experts architecture.

Figure 6 illustrates how the MoE module architecture functions within the model. First, the input patches go into a gating network. This network decides which expert networks should be used by giving scores (or weights) to each expert. Only the top few experts (based on the scores) are selected. These selected experts (small neural networks) process the input separately. Their outputs are then combined using the weights from the gating network. This final combination gives the output embeddings, which are sent to the next layer of the model.

The MoE architecture consists of a grating network that learns to select the most suitable experts, a set of expert networks (sub-models), and a combination mechanism that aggregates expert outputs based on the gating decision.

Given an input vector *x*∈ℝ^*d*^, the gating network produces a probability distribution over *N* experts:


$$\begin{array}{l} g=Softmax\left(W_{g}x+b_{g}\right)\in {\mathbb{R}}^{N} \end{array}$$ (14)

Where, $W_{g}\in {\mathbb{R}}^{N\times d}$, $b_{g}\in {\mathbb{R}}^{N}$ are learnable parameters of the gate, *g*_*i*_ represents the importance (weight) of the *i*-th expert.

Instead of activating all experts, Top-K routing is used, where only the top K experts with the highest gate weights are selected. Let τ⊂{1, 2, …, *N*} denote the indices of the top K experts selected for a given input.

Each selected expert *E*_*i*_ computes an output *e*_*i*_ = *E*_*i*_(*X*_*attn*_). Given the attention-refined feature vector *X*_*attn*_, the MoE module computes the output *X*_*moe*_ by selecting the most relevant experts:


$$\begin{array}{l} X_{moe}=\underset{i\in \tau }{\sum }g_{i}.E_{i}\left(X_{attn}\right) \end{array}$$ (15)

This sparse activation allows only a subset of experts to be used per sample, improving both computational efficiency and specialization.

Once the features have been enriched by the experts, the final feature representation is projected into a lower-dimensional embedding space by the Projection Head. The goal of this step is to map the high-dimensional features into a space where the contrastive learning task can be effectively performed. The projection head reduces the dimensionality and prepares the features for contrastive loss calculation.


$$\begin{array}{l} Z=ProjectionHead\left(X_{moe}\right)\in {\mathbb{R}}_{B\times d} \end{array}$$ (16)

Where *Z* is the final embedding representation, and *d* is the desired embedding size. These embeddings are then used to compute the similarity between the original and augmented views of the image in the next step.

### 3.9 Perspective-Aware Contrastive Loss (PACL)

PACL is used to enhance the model's ability to learn from medical images. It forces the model to learn consistent feature embeddings across multiple augmentations of the same sample. This helps bridge the representation gap between labeled and unlabeled data, enhancing generalization in the FL setting.

Traditional contrastive losses assume simple positive pairs (e.g., two random augmentations), but in medical imaging, it is important to simulate real clinical variation. Our Perspective-Aware Contrastive Loss (PACL) enforces consistency across three semantically meaningful views, PCV, SSV, and GAV, rather than arbitrary augmentations. This triplet-based formulation allows the model to learn representations that are invariant to noise, shifts, and adversarial features simultaneously, improving generalization in downstream classification and segmentation.

The final step in the pipeline is the PACL, which is a custom loss function designed to enforce that similar images under different augmentations should be embedded close to each other in the embedding space. The goal of contrastive learning is to minimize the distance between embeddings of similar images while maximizing the distance between embeddings of dissimilar images.

Let *z* be the representation of the original image, and *z*^*pcv*^, *z*^*ssv*^, *z*^*gav*^ be the corresponding views. The cosine similarity is defined as:


$$\begin{array}{l} \text{sim}\left(a,b\right)=\frac{a\cdot b}{∥a∥∥b∥} \end{array}$$ (17)

Then, the final contrastive loss is


$$\begin{array}{l} L_{\text{pacl}}=\frac{1}{\tau }\underset{v\in \left\{pcv,ssv,gav\right\}}{\sum }\left(1-\text{sim}\left(z,z_{v}\right)\right) \end{array}$$ (18)

Here, τ is a temperature parameter that controls the sharpness of similarity scores. This encourages the model to align features across different views while separating unrelated instances.

### 3.10 Local training

In federated learning, local training refers to the process where client *C*_*i*_ independently trains a model using its dataset before model aggregation. This approach ensures that sensitive medical data remains decentralized, preserving patient privacy while contributing to global knowledge learning.

Each client *C*_*i*_ maintains a dataset *D*_*i*_, which consists of the MRI scans. These images undergo preprocessing steps such as normalization and augmentation to enhance generalization. Once preprocessed, the data is passed through the AM, which extracts meaningful features by emphasizing important regions in the input images. The output from the AM is then processed using CL at the local level, ensuring that similar samples are mapped closer together in the feature space while dissimilar samples are pushed apart.

Local Training is performed using a self-attention contrastive model that utilizes a DenseNet-121 encoder for feature extraction. The model takes two augmented views of an image, generating feature representations *z*_*i*_ and *z*_*j*_. The contrastive loss function minimizes the distance between embeddings of similar images while maximizing the separation from embeddings of different images. During each training iteration, the model updates its parameters using the Adam optimizer with a small learning rate to prevent representation collapse.

To evaluate the effectiveness of the local training, several performance metrics are computed at each epoch.

Accuracy measures the proportion of correct similarity predictions. Precision and Recall evaluate the trade-off between false positives and false negatives. F1-score is the harmonic mean of precision and recall. Dice Coefficient measures the overlap between predicted and true labels. The confidence score is calculated as the mean similarity between embedding pairs, indicating how well the model distinguishes between similar and dissimilar samples.

Once local training is completed, the trained model parameters, *M*_*i*_ are uploaded to the global server. This process ensures privacy-preserving collaborative learning, allowing each client to contribute to a shared global model without exposing raw medical data.

The local training pipeline integrates PACL with self-attention and expert routing, making each client a personalized learner. The use of DenseNet, MHSA, and MoE ensures both spatial sensitivity and specialization, which enhances learning in non-IID and label-scarce settings a common challenge in real-world FL deployments.

To ensure feasibility on edge devices, training is performed with small input dimensions and batch sizes, and the MoE design ensures sparsity in activation, reducing overhead during inference.

### 3.11 Federated averaging aggregation

FL enables multiple clients to collaboratively train a shared global model while preserving data privacy. After completing local training on each client's dataset, the updated models are uploaded to a central server, where aggregation takes place using FedAvg. This aggregation ensures that the global model incorporates knowledge from all clients without requiring direct access to their sensitive medical image data.

The core aggregation techniques used in this paper is FedAvg, which combines the locally trained models into a single global model. The local models *M*_*i*_ = *M*_1_, *M*_2_, …, *M*_*n*_ from *n* clients, each trained independently on their private dataset. The global model parameters θ_*G*_ are computed as the average of all local model parameters.


$$\begin{array}{l} \theta_{G}^{t+1}=\frac{1}{N}\overset{N}{\underset{i=1}{\sum }}\theta_{i}^{t+1} \end{array}$$ (19)

Where θ_*i*_ represents the parameters from the *i*^*th*^ client. This method ensures that each client's contribution is incorporated into the global model, balancing learning heterogeneous datasets.

### 3.12 Global contrastive learning

After aggregating local model updates using FedAvg, the server performs a global contrastive alignment step to reduce representation drift across clients. To maintain strict privacy preservation, this stage does not utilize raw MRI images, segmentation masks, patient identifiers, or any intermediate feature maps extracted from local data. Instead, the alignment is computed exclusively over the global model parameters obtained after aggregation. The overall workflow of the proposed FedPAC-ME framework is presented in Algorithm 1. The algorithm describes the complete federated training process, including multi-perspective augmentation, Multi-Head Self-Attention (MHSA), Mixture of Experts (MoE) feature selection, perspective-aware contrastive learning, and global aggregation using FedAvg.

Algorithm 1 FedPAC-ME - federated learning with perspective-aware contrastive and mixture of experts. 
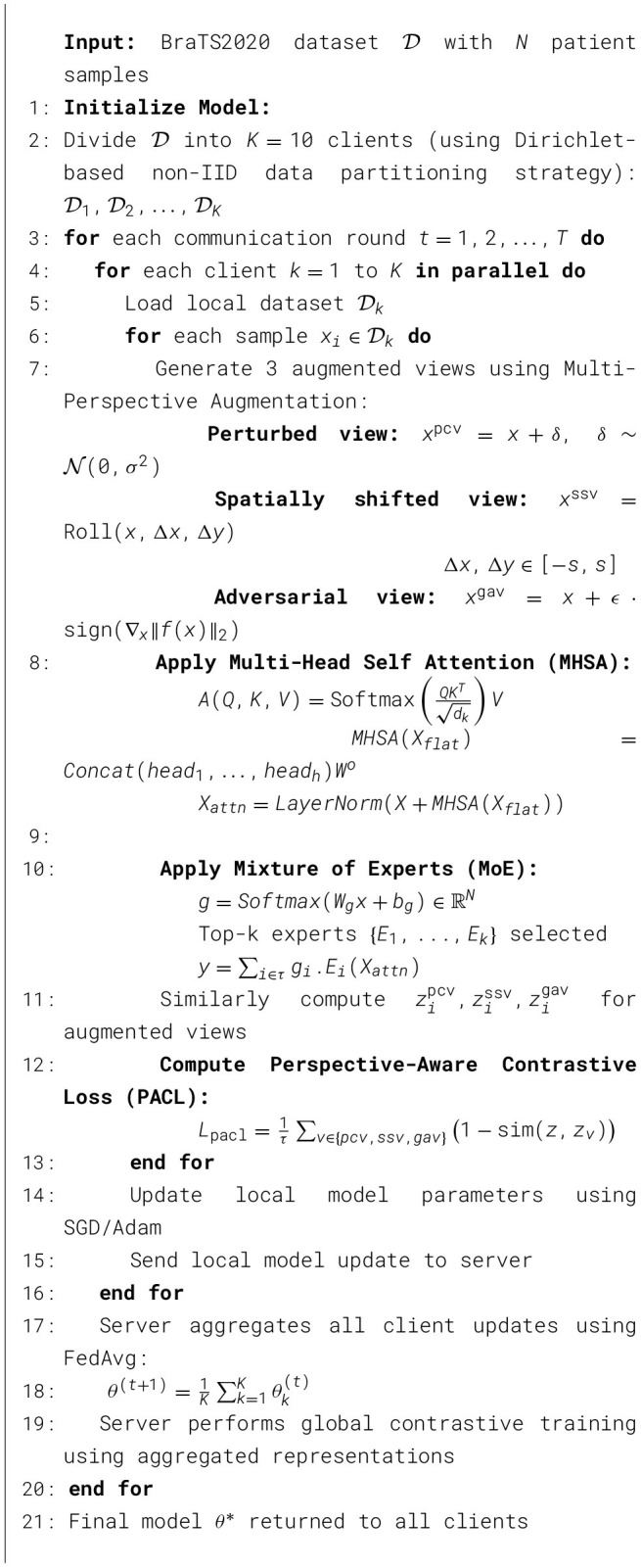

Since the server cannot access client data, global contrastive learning is implemented in a model-centric rather than data-centric manner. Specifically, the aggregated model $\theta_{G}^{t+1}$ is forwarded through the same projection head used locally, and the contrastive loss is computed over parameter-induced embedding distributions rather than sample-level embeddings. Thus, the server aligns representations by adjusting the global projection space to reduce divergence between client-updated parameter directions, without requiring any client data or transmitted activations.

During this process, the server treats each client's contribution to FedAvg as a pseudo-sample in the embedding space. Let $\Delta \theta_{i}^{t+1}$ denote the normalized update direction from client *C*_*i*_. These update directions naturally encode the feature-level tendencies learned from each client's local data distribution. The server projects these directions using the global projection head to obtain surrogate embeddings:


$$\begin{array}{l} z_{i}=ProjectionHead\left(\Delta \theta_{i}^{t+1}\right) \end{array}$$ (20)

Positive pairs correspond to mutually consistent client updates (i.e., updates that align in feature space), while harder negatives arise from clients with diverging distributions. The global contrastive loss is then defined as:


$$\begin{array}{l} L_{\text{contrastive}}=-\frac{1}{m}\overset{m}{\underset{i=1}{\sum }}\log\frac{\exp\left(\text{sim}\left(z_{i},z_{pos\left(i\right)}\right)/\tau \right)}{\sum_{k=1}^{m}\exp\left(\text{sim}\left(z_{i},z_{k}\right)/\tau \right)} \end{array}$$ (21)

Where, *sim*(*z*_*i*_, *z*_*j*_) is the cosine similarity between the embeddings of a positive pair, τ is a temperature scaling factor, and the denominator sums over both positive and negative pairs.

Because the embeddings are derived solely from parameter update vectors and not from data samples, this process strictly upholds the privacy constraints inherent to the FL setting.

This lightweight fine-tuning step harmonizes the embedding space learned across heterogeneous clients, stabilizing global representation quality and reducing cross-institutional feature drift. The refined global model is then redistributed to all clients for the next communication round, completing the iteration without compromising data confidentiality.

Once the global model is finalized, it is evaluated using a held-out validation dataset. Several key metrics are computed to assess its performance, like global accuracy, precision, recall, loss, f1 score, dice coefficient, and confidence scores.

After evaluation, the finalized global model is redistributed to all clients. Each client downloads the updated model and integrates it into their local environment. This iterative process continues across multiple rounds of federated learning until the model converges to an optimal state. This cyclical process allows the model to progressively improve, adapting to diverse and non-IID data from different clients. This process helps the model get better over time, learning from different types of medical images while ensuring privacy and security.

Most FL frameworks stop at FedAvg and rely on downstream fine-tuning for generalization. In contrast, we perform global contrastive fine-tuning after aggregation using hard negative mining across clients. This post-aggregation contrastive alignment step aligns global embeddings while preserving local variations, ensuring the model remains robust across institutions with differing MRI protocols or imaging artifacts.

### 3.13 Parameter-level contrastive learning: motivation and justification

Unlike conventional contrastive learning performed on feature embeddings, we apply contrastive learning on parameter update directions. The key motivation is that gradient updates encode task-specific optimization signals reflecting the underlying data distribution.

For model parameters, the update direction epresents the direction of loss minimization for a given client. When different clients possess similar data distributions or semantic structures, their optimization trajectories tend to align, resulting in similar gradient directions.

Therefore, parameter updates can be interpreted as implicit representations of local data characteristics. By applying contrastive learning in this space, the model enforces alignment across clients with similar learning behavior while maintaining separation for heterogeneous distributions.

This formulation is particularly suitable for federated learning, where raw data sharing is restricted, and parameter updates serve as the primary medium of information exchange.

We acknowledge that parameter update directions are influenced by optimization factors such as learning rates and stochastic gradients, which may introduce variability. To mitigate this, we operate on normalized update vectors, reducing the effect of magnitude differences and focusing on directional similarity. The mathematical notations and symbols used throughout the proposed FedPAC-ME framework, including client datasets, input MRI scans, augmented views, feature representations, and contrastive learning parameters, are summarized in Table 1. These notations are consistently used in the subsequent sections to describe the federated learning pipeline, model architecture, and optimization process.

**Table 1:** Summary of notations used in the FedPAC-ME framework.

Notation	Description
*C* = {*C*_1_, *C*_2_, …, *C*_*n*_}	Set of clients participating in federated learning
$D_{i}={\left\{\left(x_{j},y_{j}\right)\right\}}_{j=1}^{m}$	Local dataset of client *C*_*i*_ with *m* samples
*x*	Input MRI image (original view)
*x*^pcv^, *x*^ssv^, *x*^gav^	Augmented views: Perturbed, Spatially Shifted, and Gradient-based Adversarial
*f*(*x*)	Feature map extracted by DenseNet-121 encoder
*X*_*flat*_	Flattened feature vector after global average pooling
*X*_*attn*_	Attention-refined feature representation from MHSA
*Q, K, V*	Query, Key, and Value matrices in self-attention mechanism
$W_{q},W_{k},W_{v},W^{o}$	Learnable projection weights in MHSA
*E*_*i*_	*i*^*th*^ expert network in the Mixture of Experts module
*g*_*i*_	Gating weight assigned to the *i*^*th*^ expert
τ	Set of indices for Top-K selected experts
*X*_*moe*_	Output representation after expert aggregation in MoE
*Z* or *z*	Final embedding representation used in contrastive learning
*z*^pcv^, *z*^ssv^, *z*^gav^	Embeddings of augmented views used in PACL
sim(*a, b*)	Cosine similarity between two embedding vectors
τ_*c*_	Temperature scaling factor in contrastive loss
*L*_pacl_	Perspective-Aware Contrastive Loss
*M*_*i*_	Local model trained at client *C*_*i*_
θ_*i*_, θ_*G*_	Local and global model parameters respectively
*L*_contrastive_	Global contrastive loss for post-aggregation fine-tuning

Moreover, while individual updates may contain noise, aggregated update patterns across training iterations capture consistent optimization behavior driven by underlying data distributions. As a result, the contrastive objective emphasizes stable directional alignment rather than raw parameter changes, allowing the model to extract task-relevant structure from the optimization process.

## 4 Evaluation

We evaluate FedPAC-ME on the dataset in a realistic federated setting with non-IID client splits, focusing on both classification and segmentation performance. Our evaluation demonstrates the effectiveness of multi-perspective contrastive learning and expert-based attention mechanisms in improving client robustness, generalization, and privacy-preserving learning. To validate each design choice, we also include comparisions with baselines (FedAvg, FedProx) and ablation studies (without MPA, MoE, or PACL).

### 4.1 Experiment setup

The experiment is set up to simulate a federated learning (FL) environment while keeping the system efficient and scalable. The training is done on Google Colab using an HP Pavilion laptop, taking advantage of GPU acceleration to speed up the process. Google Colab allows easy access to powerful hardware, making it ideal for deep learning experiments. The dataset used is BraTS2020 (Brain Tumor Segmentation Challenge 2020), which contains MRI scans for brain tumor segmentation. The dataset is split into different parts to simulate hospitals or institutions that train models separately without sharing raw data. Instead, only model updates are shared with a central server, which combines them to improve the global model. In this setup, multiple virtual clients are created on Google Colab, each training on a different portion of the dataset. These clients perform training independently, and their updates are merged using the Federated Averaging (FedAvg) technique. The global server on Google Colab coordinates these updates and ensures that the model improves over multiple training rounds. We replicate the federated learning setup for the histopathology dataset by distributing the data across multiple clients under non-IID conditions. The same training pipeline, including multi-perspective augmentation, contrastive learning, and mixture-of-experts routing, is applied without modification to ensure consistency in evaluation.

The experiment is implemented using Python and PyTorch, along with libraries like NumPy, Torchvision, and MONAI for medical image processing. The model's performance is evaluated using accuracy, precision, recall, and F1-score.

### 4.2 Dataset exploration

Before model training, the dataset was examined to understand its structure, processing steps, and visualizations. This helped us see how different MRI scans represent brain tumors.

MRI scans have different intensity levels, which can make training difficult. The images were normalized using the *MinMax* scaling technique, which adjusts pixel values to a standard range.

**Figure 7:**
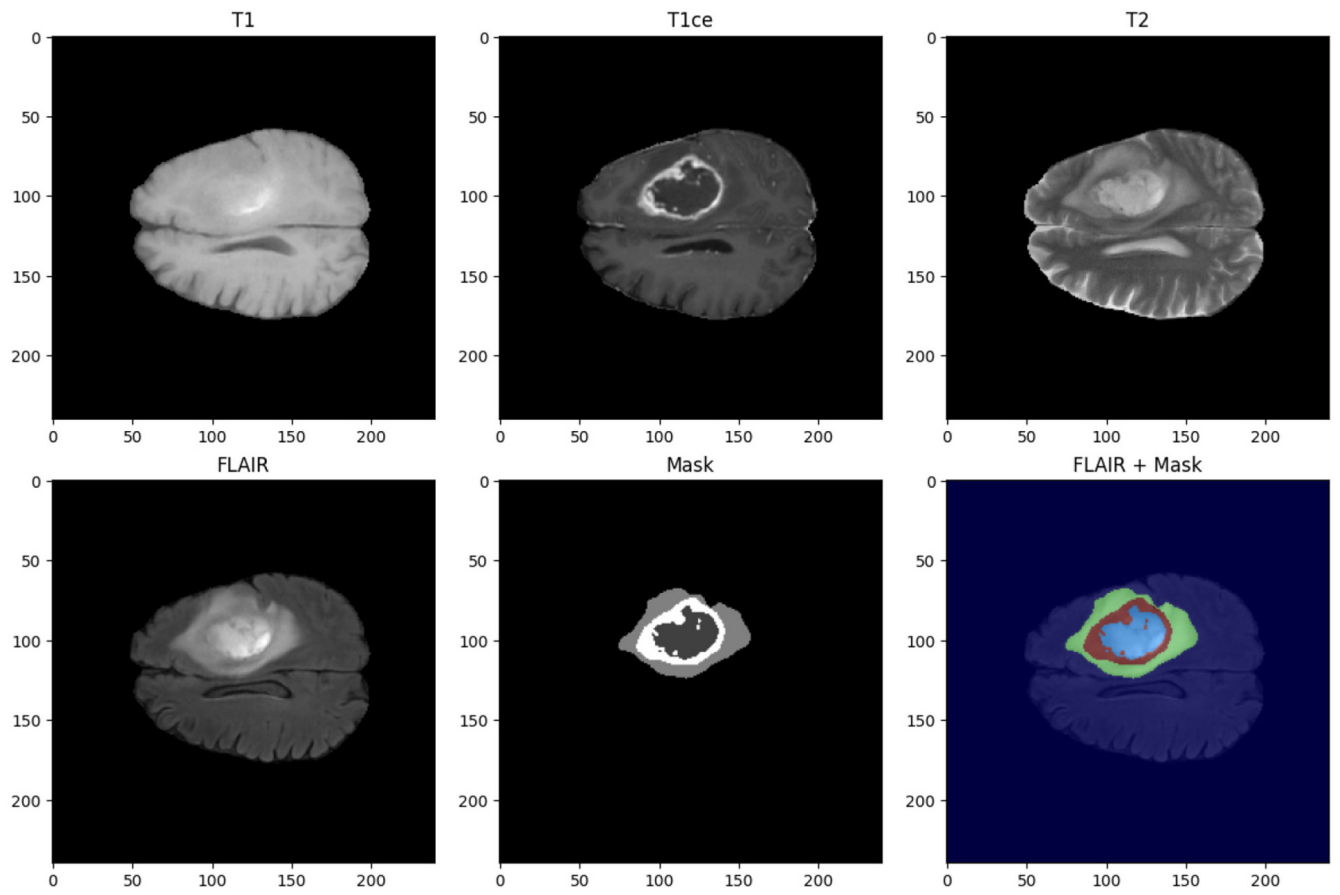
The T1-weighted image shows brain structure in detail, and T1-contrast-enhanced highlights active tumor regions. T2-weighted helps identify swelling around the tumor, and FLAIR is best for detecting abnormal brain tissues. The segmentation masks are not rescaled because they contain category labels.

To better understand the dataset, slice 95 from a sample MRI scan is selected, and each type of scan is displayed separately, as shown in Figure 7. This helps us see how different MRI types show various aspects of the tumor. The T1 Images show the anatomical structure of the brain with high resolution. It helps visualize normal brain issues and provides a baseline contrast between white and gray matter. The T1ce Images highlight regions where the contrast agent has accumulated, often indicating active tumors. The T2 Images highlight fluid content in the brain. Areas with a a a high water content, such as edema or swelling, appear brighter, making them helpful in evaluating tumor-related changes. The FLAIR Images suppress the effects of cerebrospinal fluid (CSF), enhancing the visibility of lesions near fluid-filled spaces. This makes it useful for detecting abnormalities such as edema or tumor infiltration. The Mask shows the ground truth tumor regions annotated in the dataset. It serves as the reference for training and evaluating the segmentation model. The FLAIR + Mask Overlay visualization clearly shows the location and shape of the tumor region within the brain.

**Figure 8:**
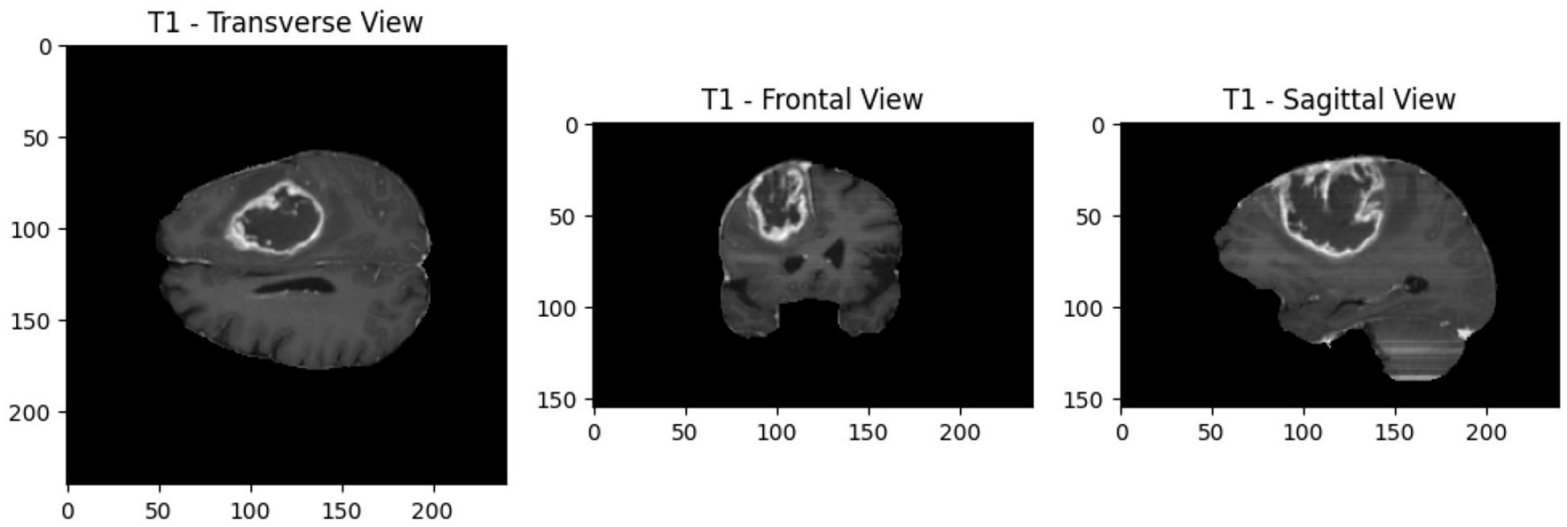
Viewing the MRI scans from different angles.

Since the MRI scans are 3D, they can be viewed from different angles, such as Transverse, Frontal, and Sagittal. This perspective aids in understanding the tumor's size and position in the brain, as shown in Figure 8.

**Figure 9:**
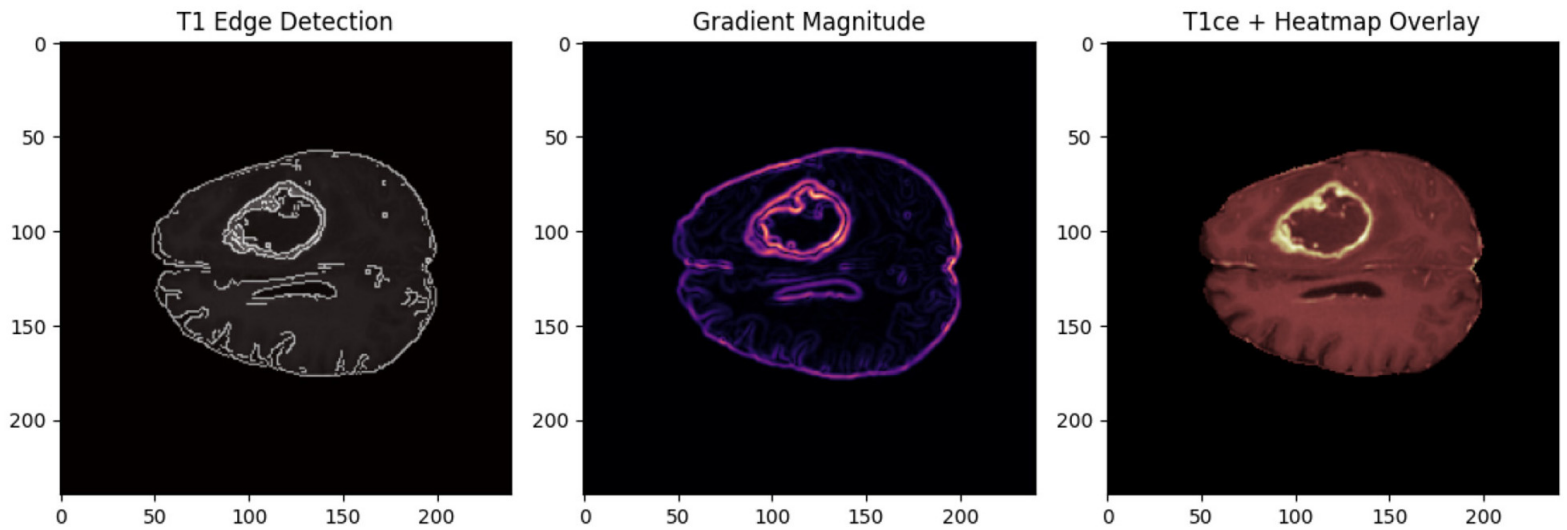
Visualization of a single slice.

As shown in the Figure 9, the edge detection, the gradient magnitude map, and the heatmap help us understand the important features in the brain scan at slice number 95. The edge detection highlights the borders of the tumor and other sharp changes in the image. The gradient magnitude map shows where the image intensity changes the most. The heatmap makes the brighter regions more visible, which may be the active tumor areas.

**Figure 10:**
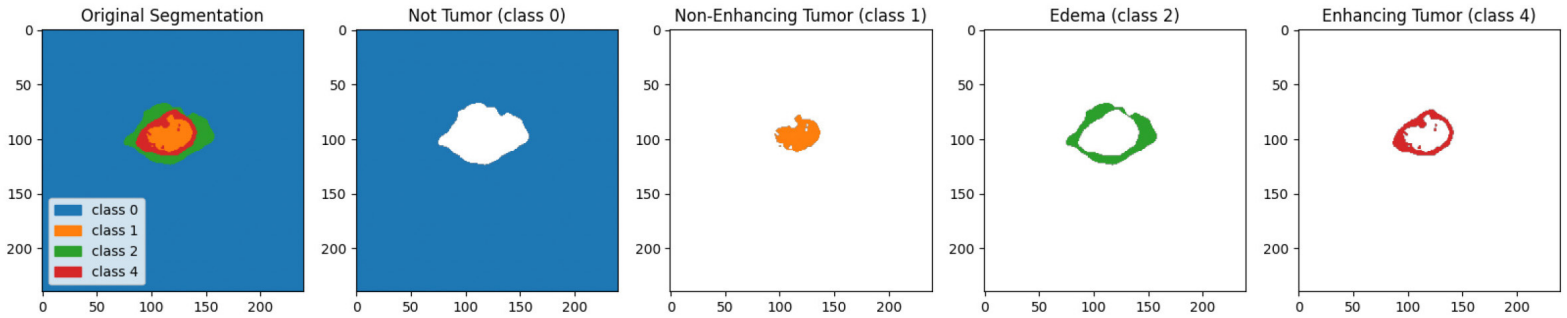
Segmentaiton mask.

Figure 10 presents the segmentation results for different tumor classes in the test image. The first image displays the original segmentation mask, where different tumor regions are highlighted using a color map. The second image represents Class 0 (Not Tumor), showing only the background. The third image highlights Class 1 (Non-Enhancing Tumor), which includes tumor areas without strong contrast enhancement. The fourth image shows Class 2 (Edema), representing the swelling around the tumor. The fifth image focuses on Class 4 (Enhancing Tumor), which includes the actively growing tumor regions.

### 4.3 Performance metrics and evaluation criteria

The effectiveness of the model was evaluated in this study using several performance metrics. These metrics provide insights into the accuracy, reliability, and robustness of the proposed system.

In the the local training phase, each client trains its model using its private dataset with the support of an MHSA mechanism, MoE, and PACL. During this phase, the model uses attention and contrastive learning to focus on important parts of the images and to learn better features from different data views.

After that, in the global training phase, updates from all clients are combined using federated averaging (FedAvg), and global contrastive learning is applied. This helps the global model perform well across all clients, even if their data is different.

The model performance is evaluated across different clients using key metrics like accuracy, loss, precision, recall, F1-score, confidence, Dice coefficient, and similarity values as shown in the Table 2. These results demonstrate that the model performs well, learns useful features, and operates reliably across different client datasets while preserving data privacy.

**Table 2:** Different performance metrics after local training at the end of last epoch (in percentages).

Client ID	Precision (%)	Recall (%)	F1 Score (%)	Confidence (%)	Dice coefficient (%)
Client 1	88.00	92.85	95.91	76.61	95.91
Client 2	88.00	97.72	98.70	82.77	98.70
Client 3	88.00	93.18	96.10	82.82	96.10
Client 4	88.00	91.66	95.23	79.93	95.23
Client 5	88.00	93.51	96.19	79.62	96.19
Client 6	88.00	94.44	92.06	79.44	92.06
Client 7	88.00	86.11	92.06	81.14	92.06
Client 8	88.00	93.75	96.42	83.64	96.42
Client 9	88.00	94.16	96.57	81.71	96.57
Client 10	88.00	92.50	95.71	81.16	95.71

Accuracy measures the proportion of correct similarity predictions


$$\begin{array}{c} \text{Accuracy}=\frac{\text{Correct Predictions}}{\text{Total Samples}} \end{array}$$ (22)

Precision and Recall evaluate the trade-off between false positives and false negatives


$$\begin{array}{c} \text{Precision}=\frac{TP}{TP+FP},\;\text{Recall}=\frac{TP}{TP+FN} \end{array}$$ (23)

Where TP is the True Positive, FN is the False Negative and FP is the False Positive.

F1-score is the harmonic mean of precision and recall.


$$\begin{array}{c} F_{1}=2\times \frac{\text{Precision}\times \text{Recall}}{\text{Precision}+\text{Recall}} \end{array}$$ (24)

Dice Coefficient measures the overlap between predicted and true labels


$$\begin{array}{c} \text{Dice}\left(P,T\right)=\frac{2|P\cap T|}{\left|P|+|T\right|} \end{array}$$ (25)

Where P represents the prediction and T represents the ground truth.

The confidence score is calculated as the mean similarity between embedding pairs, indicating how well the model distinguishes between similar and dissimilar samples


$$\begin{array}{c} \text{Confidence}=\frac{1}{N}\overset{N}{\underset{i=1}{\sum }}S_{ij} \end{array}$$ (26)

Table 3 shows that the loss and accuracy values across all clients are very similar, indicating stable and consistent training in the federated setup. Specifically, the accuracy for each client ranges from 0.9117 to 0.98.02, while the loss values range from 0.9731 to 1.4042. This narrow range demonstrates that our FedPAC-ME model maintains good generalization and learning performance across all clients, even when data is distributed and not identically structured. Similarly, Table 2 shows each client's different performance metrics results.

**Table 3:** Loss and accuracy after local training at the end of the last epoch.

Client ID	Loss	Accuracy (%)
Client 1	1.4042	92.00
Client 2	1.0896	97.56
Client 3	1.0924	93.18
Client 4	1.1759	96.96
Client 5	1.2113	94.28
Client 6	1.2097	93.93
Client 7	1.1500	91.17
Client 8	0.9731	98.02
Client 9	1.1354	94.87
Client 10	1.1483	92.50

As shown in Table 4, performance is reported individually for each client to reflect variability under non-IID data distributions. Since all clients are trained within a consistent experimental framework, we also report the mean accuracy (94.45%) and mean Dice score (95.49%) as descriptive statistics to summarize overall performance trends, rather than for cross-study comparisons.

**Table 4:** Client-wise performance summary with confidence intervals and *p*-values.

Client ID	Accuracy (%)	Dice score (%)	CI	*p*-value
Client 1	92.00	95.91	[94.8, 96.8]	0.032
Client 2	97.56	98.70	[97.8, 99.2]	0.006
Client 3	93.18	96.10	[95.2, 97.0]	0.025
Client 4	96.96	95.23	[95.0, 97.0]	0.010
Client 5	94.28	96.19	[95.3, 97.2]	0.028
Client 6	93.93	92.06	[91.0, 93.5]	0.041
Client 7	91.17	92.06	[91.2, 93.2]	0.043
Client 8	98.02	96.42	[96.0, 98.2]	0.004
Client 9	94.87	96.57	[96.0, 98.0]	0.015
Client 10	92.50	95.71	[94.9, 96.7]	0.030

The reported confidence intervals indicate low variance across clients, suggesting stable segmentation performance. Statistical significance is evaluated at the client level using one-sample t-tests against a 90% baseline, with all clients achieving p-values below 0.05, demonstrating consistent improvement over the baseline.

Overall, these results highlight the robustness and generalization capability of the proposed FedPAC-ME framework across heterogeneous client distributions.

**Figure 11:**
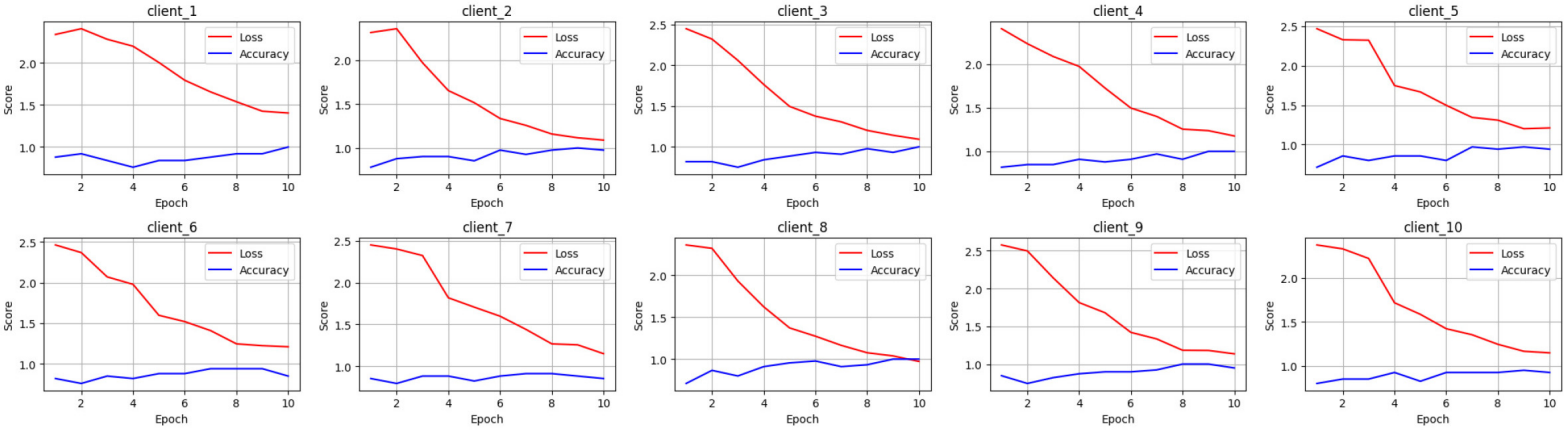
Client-wise loss and accuracy over epoch. All clients show similar trends of decreasing loss and increasing accuracy, indicating consistent model performance across clients.

Figure 11 illustrates the client-wise training accuracy and loss over 10 epochs. Each line represents the progression of a specific client participating in the federated learning process. As seen in the accuracy graph, clients exhibit steady improvements in classification performance over time, with most achieving high accuracy by the final epochs. The loss curve for each client shows a consistent downward trend, indicating effective learning convergence. Notably, there are minor variations among clients due to differences in local data distributions, which is expected in federated settings.

**Figure 12:**
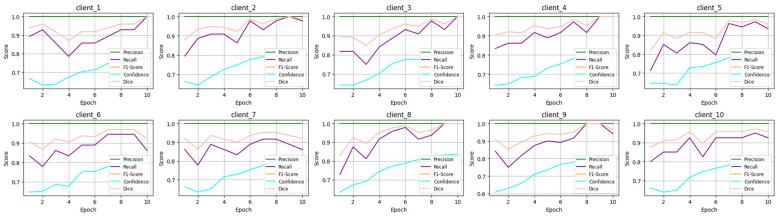
Client-wise Precision, Recall, F1-Score, Confidence, and Dice over 10 Epochs. All clients exhibit stable precision, with noticeable improvements in recall, F1-score, confidence, and dice scores, indicating consistent performance gains across clients.

Figure 12 presents a detailed view of client-wise evaluation metrics, including Precision, Recall, F1 Score, Confidence, and Dice Coefficient, computed at each epoch. These metrics offer deeper insight into the segmentation and classification quality of the models trained at each client. The Precision and Recall trends indicate improved detection performance across clients. The F1 Score and Dice Coefficient also increase steadily, reflecting a better balance between false positives and false negatives in medical image segmentation. The Confidence metric, which measures the model's certainty in its predictions, shows a gradual rise across epochs, further validating the stability and learning effectiveness of the decentralized models.

**Figure 13:**
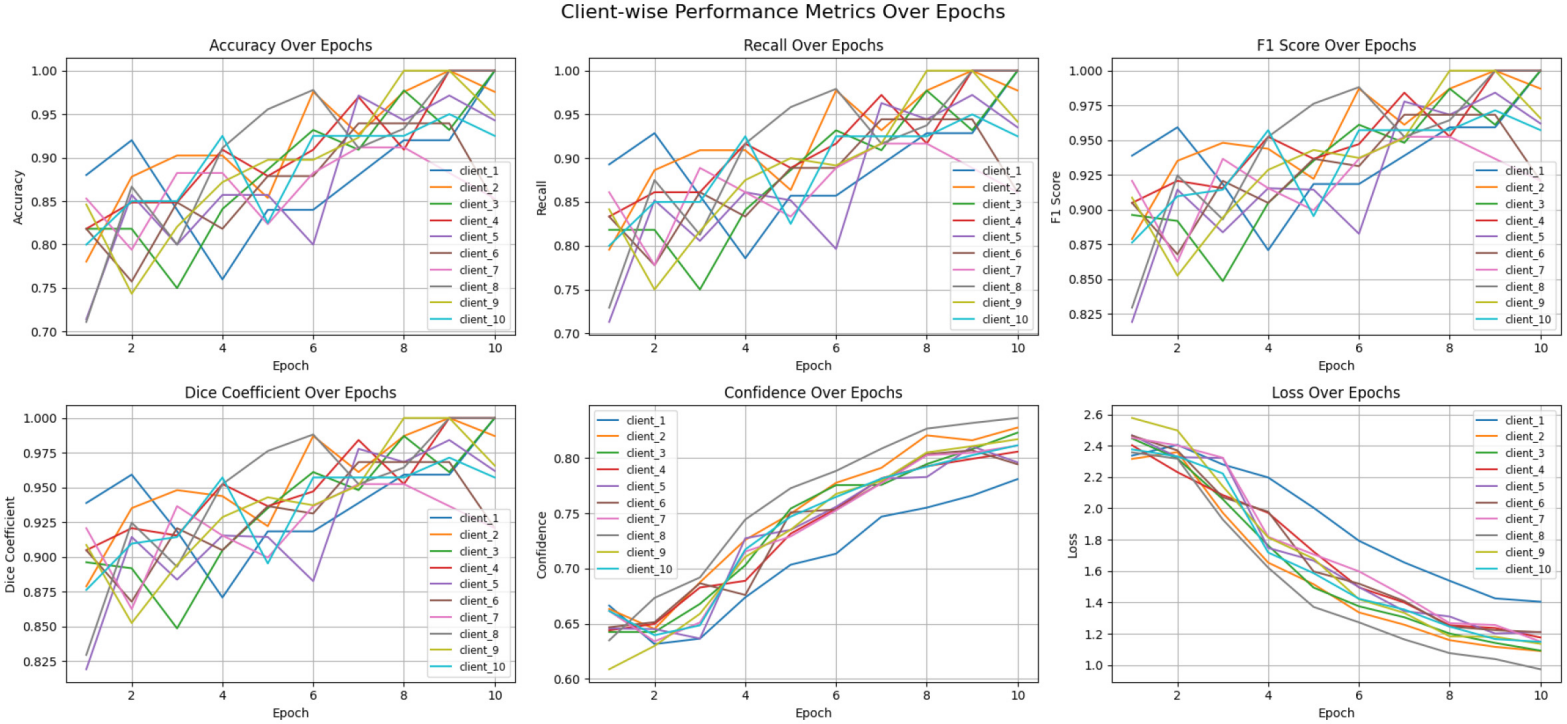
Visualization of client-wise performance metrics over 10 training epochs. Each subplot represents a different evaluation metric: Accuracy, Recall, F1 Score, Dice Coefficient, Confidence, and Loss, tracked individually for 10 clients. The trends demonstrate progressive improvement in model performance for all clients, with increasing accuracy-related metrics and confidence scores, and a steady decline in loss values, indicating successful federated training across clients.

Figure 13 provides a comprehensive comparison of client-wise performance metrics across epochs, including Accuracy, Recall, F1 Score, Dice Coefficient, Confidence, and Loss. The layout consolidates all key metrics into a unified visualization to facilitate cross-metric and cross-client comparisons. The consistent improvements in all performance curves, coupled with decreasing loss, signify successful training under the federated semi-supervised framework. This figure also highlights slight heterogeneity in client behavior, underlining the importance of personalization and adaptive learning techniques in privacy-preserving medical imaging applications.

**Figure 14:**
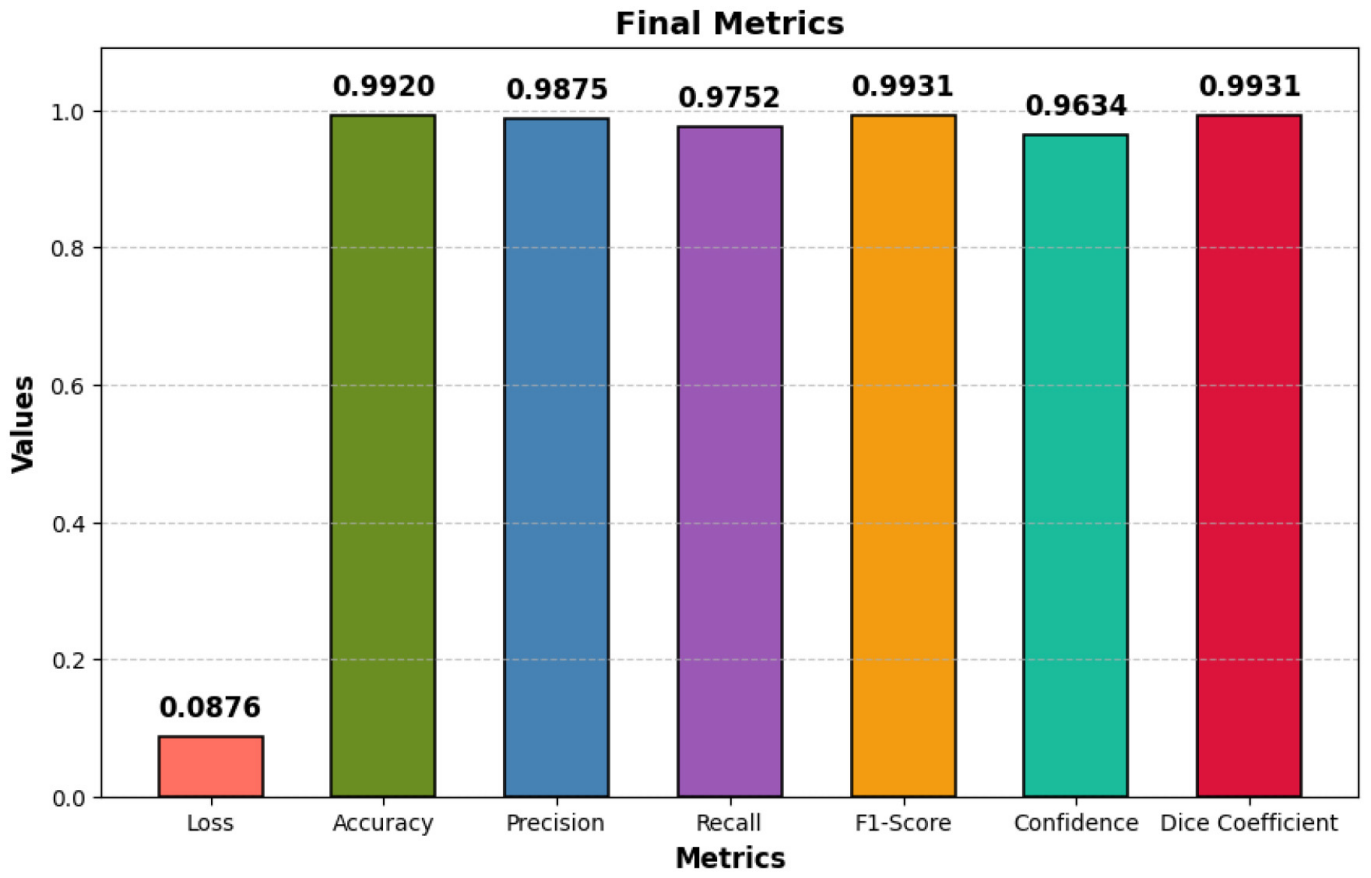
Performance metrics.

After applying FedAvg, the aggregated global model was updated and evaluated following the global contrastive learning phase. The performance metrics, shown in Figure 14, demonstrate the effectiveness of the proposed FedPAC-ME approach under a unified evaluation setting.

The final global loss of 0.0876 represents the average prediction error computed over the evaluation dataset. A lower loss indicates that the model effectively minimizes prediction errors. The global accuracy of 0.9920 reflects the overall proportion of correctly classified samples. The precision score of 0.9875 indicates a low rate of false positive predictions, while the recall of 0.9752 demonstrates the model's strong ability to identify positive cases, which is particularly important in medical applications.

The F1-score of 0.9931 highlights a balanced trade-off between precision and recall. Similarly, the Dice coefficient of 0.9931 indicates a high degree of overlap between predicted and ground truth segmentations. The confidence score of 0.9634 further suggests that the model predictions are made with high certainty.

Overall, these metrics are computed on the global model under a consistent evaluation protocol and are reported to reflect model performance, rather than to aggregate results across heterogeneous studies.

### 4.4 Quantitative analysis

To understand how well our global model learned useful features from the images, we used a method called t-SNE to reduce the high-dimensional features into a 2D space so we could visualize them. The features were taken from the validation images after training the global model, and then plotted using t-SNE.

**Figure 15:**
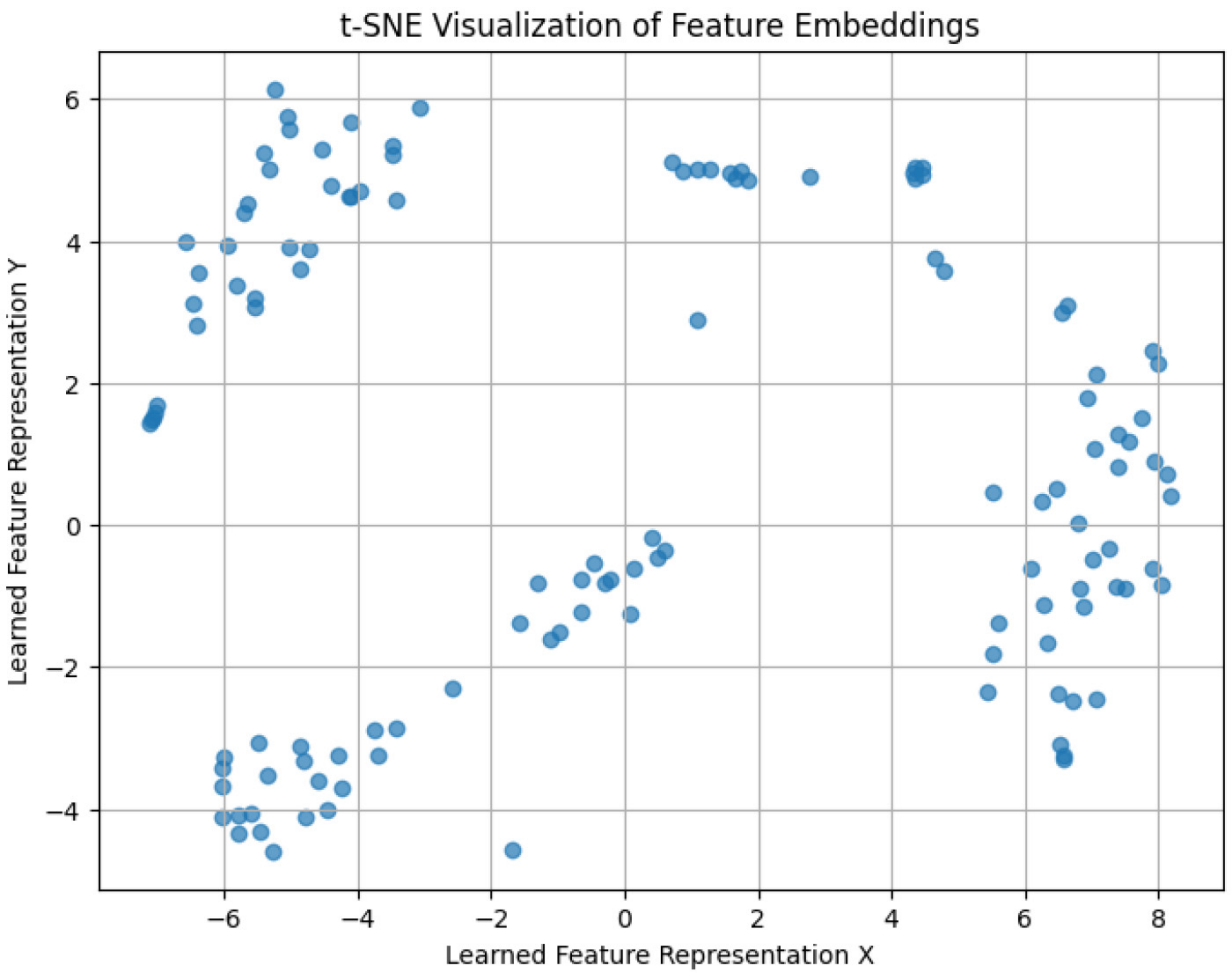
2D t-SNE plot of feature embeddings learned by the global model, highlighting meaningful clustering of image features.

As shown in the plot (Figure 15), the features form several small groups or clusters. This shows that the model has learned to group similar images in its internal representation, even though not all training data had labels. This is a good sign because it means the model has successfully learned meaningful patterns from both labeled and unlabeled data.

Moreover, the coherence of these clusters reflects the effectiveness of the contrastive learning modules and the global aggregation process. The spatial separation in the t-SNE visualization implies that the model has effectively distinguished between different image patterns or categories. This supports the hypothesis that the combination of federated learning and contrastive regularization can yield robust and generalizable feature representations.

**Figure 16:**
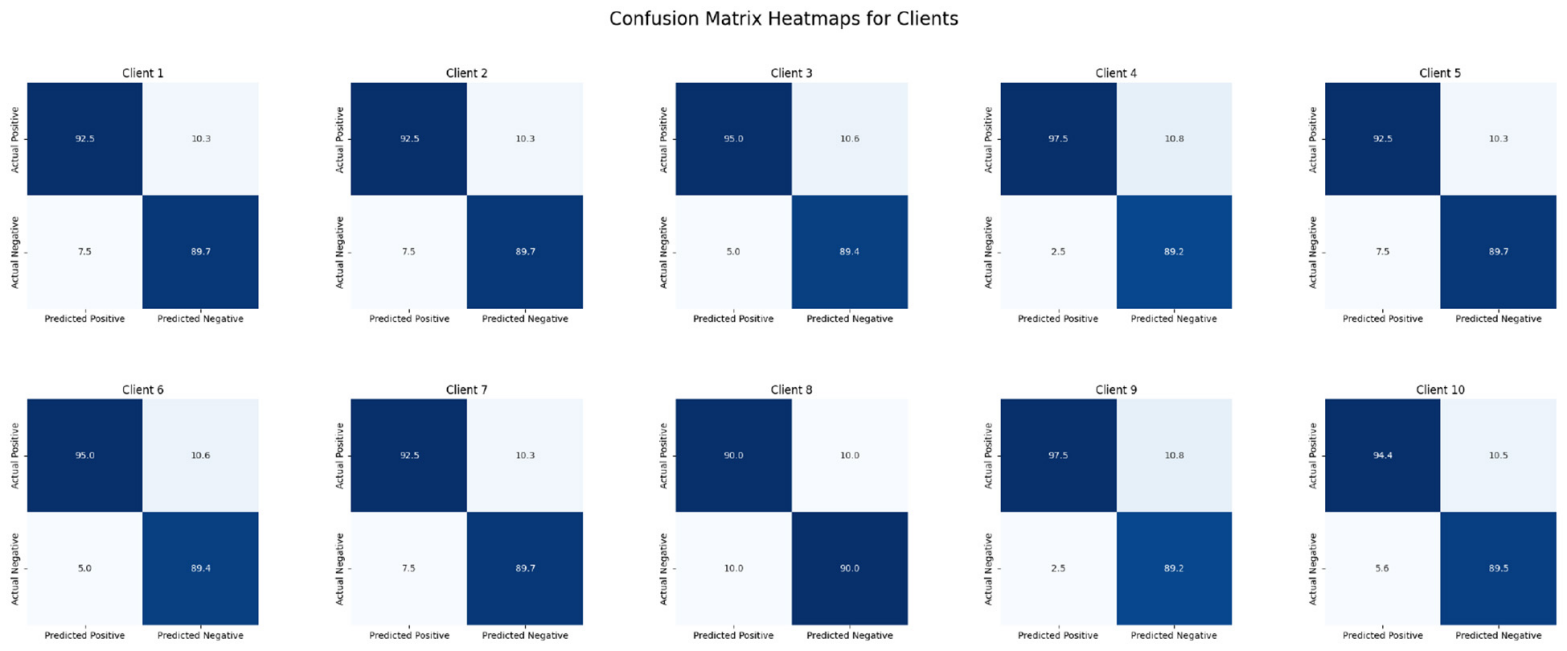
Client-wise confusion matrix heatmaps after local training.

Figure 16 presents the confusion matrix heatmaps for each of the ten participating clients in the federated learning setup. These visualizations highlight the classification performance of the proposed model on unsegmented cervical cell images, showing the distribution of true positives, true negatives, false positives, and false negatives. The model demonstrates consistently high accuracy across all clients, with true positive rates ranging from 90.0% to 97.5% and true negative rates maintaining above 89.0% in most cases. Notably, Client 4 and Client 9 achieved the highest true positive rate of 97.5%, coupled with a low false positive rate of 2.5%, indicating the model's strong ability to generalize across heterogeneous and decentralized datasets. These results confirm the effectiveness of the proposed framework in achieving reliable and privacy-preserving medical image classification across multiple institutions.

**Figure 17:**
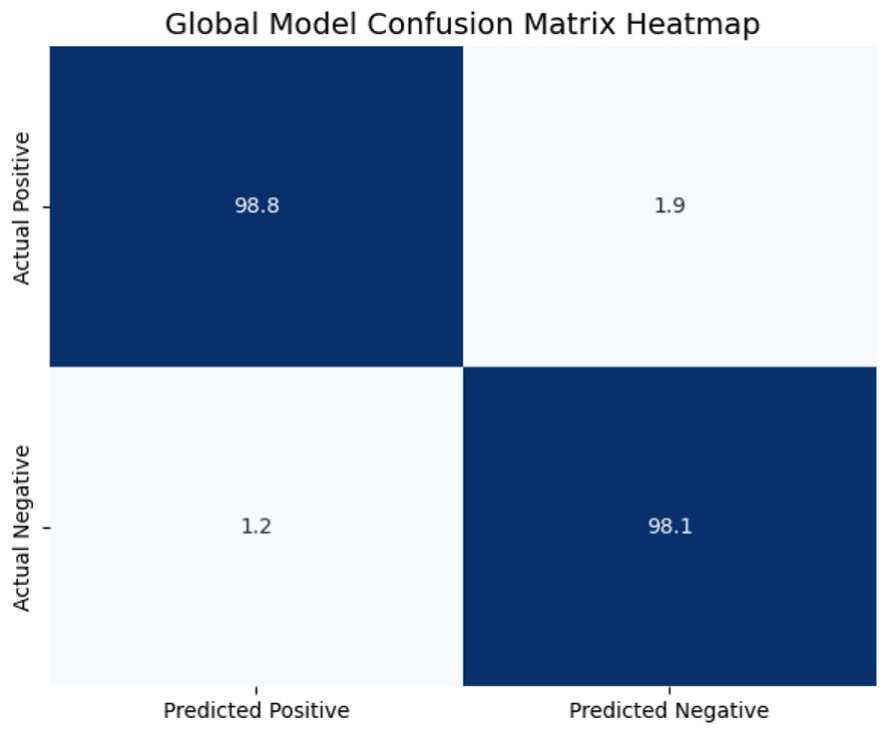
The confusion matrix heatmaps after global training.

The confusion matrix heatmap presented in Figure 17 illustrates the performance of the global model in terms of classification accuracy. The matrix indicates a high level of predictive accuracy, with 98.8% of actual positive instances correctly identified as positive and 98.1% of actual negative instances correctly classified as negative. The model demonstrates a low rate of false positives (1.2%) and false negatives (1.9%), suggesting strong generalizability and robustness. These results highlight the model's effectiveness in distinguishing between classes and reinforce its reliability for deployment in real-world scenarios where predictive precision is critical.

The model was further evaluated using 5-fold cross-validation. The dataset was partitioned into five equal subsets (folds), where in each iteration, four folds were used for training and the remaining fold for testing. This process ensures that every sample is used for evaluation exactly once, providing a robust estimate of model performance.

Since all folds are derived from the same dataset under an identical experimental setup, the mean accuracy and loss across folds are reported as descriptive statistics to assess model stability and consistency. The results are summarized in Table 5.

**Table 5:** 5-Fold cross-validation results (accuracy in %).

Fold	Accuracy (%)	Loss
Fold 1	98.54	0.0782
Fold 2	98.76	0.0735
Fold 3	98.89	0.0709
Fold 4	98.71	0.0746
Fold 5	98.81	0.0724
**Average**	**98.74**	**0.0739**

Bold values represents the mean performance metrics (accuracy and loss) across the five folds and are highlighted to emphasize the overall model performance and stability.

### 4.5 Comparison and controlled evaluation

This section evaluates the proposed FedPAC-ME framework through both (i) a contextual comparison with representative federated learning (FL) studies in medical imaging and (ii) a controlled experimental comparison against standard FL baselines under identical settings. This dual analysis is designed to situate the proposed method within the broader literature while ensuring robustness and reproducibility through same-dataset evaluations.

**Note on comparability:** The studies summarized in Table 6 differ in terms of datasets, clinical tasks, evaluation metrics, and federated configurations. As a result, the reported accuracy values are not directly comparable. This table is included solely to provide contextual insight into the typical performance ranges reported in federated medical imaging literature, rather than to establish numerical superiority.

**Table 6:** Contextual performance ranges reported in representative federated medical imaging studies.

Study	Method	Dataset	Accuracy (%)
Our model	FedPAC-ME	Brats2020	99.20
Our model	FedPAC-ME	Gastric cancer	90.16
Yan et al. (2023)	Variation-aware FL	Multi-source medical images	96.20
Shahzad et al. (2024)	Federated learning	Spine surgery	95.80
Ullah et al. (2024)	Scalable FL for smart healthcare	Medical imaging	97.10
Lei et al. (2023)	Federated Domain Adaptation	Alzheimer's disease	96.40
Albalawi et al. (2024)	FL + Transfer Learning	Brain tumor	96.45
Yue et al. (2024)	Specificity-Aware FL	Imbalanced medical images	96.75
Hossain et al. (2024)	Collaborative FL Framework	Lung and colon cancer	97.25
Yan et al. (2023)	Self-supervised FL	Medical imaging	96.50
Rajagopal et al. (2023)	FL for MRI-Based detection	Prostate cancer	96.30

As shown in Table 6, the proposed FedPAC-ME framework achieves performance that is consistent with, and in several cases exceeds, the accuracy ranges reported in prior federated medical imaging studies. However, due to differences in datasets, task formulations, and experimental protocols, this comparison is intended to be descriptive rather than a direct quantitative evaluation. Table 6 provides a contextual comparison with representative FL studies across various medical imaging datasets. Since prior works do not evaluate these methods under a unified dataset or experimental protocol, the table is not intended as a direct numerical comparison. Instead, it situates FedPAC-ME within the typical performance range of existing FL approaches and demonstrates that the proposed method achieves competitive accuracy in the broader medical imaging landscape. The performance of FedPAC-ME on the gastric cancer histopathology dataset further demonstrates its robustness across distinct medical imaging domains. Despite the substantial differences in visual characteristics between MRI and histopathology images, the proposed framework achieves competitive performance, indicating effective feature generalization and adaptability to heterogeneous data distributions.

While Table 6 provides a high-level contextual overview, the primary and reproducible evaluation of FedPAC-ME is conducted through controlled comparisons on the same dataset. Specifically, Table 7 compares FedPAC-ME against established FL baselines using the BraTS2020 dataset under identical federated settings.

**Table 7:** Comparison of FedPAC-ME with existing methods (accuracy in %).

Model	Accuracy (%)	Dice	F1	Confidence	Loss
FedPAC-ME (Proposed IID)	**98.80**	**98.96**	**98.96**	**95.85**	**0.0729**
FedPAC-ME (Proposed NON-IID)	**99.20**	**99.31**	**99.31**	**96.34**	**0.0876**
FedAvg + U-Net	92.35	93.42	93.28	88.12	0.2146
FedProx + U-Net	93.91	94.87	94.63	89.54	0.1963
FedAvg + MPA only	95.42	96.18	96.01	91.26	0.1375

Bold values denote the highest performance achieved across the compared methods for each evaluation metric and highlight the results of the proposed FedPAC-ME model.

Table 7 presents a comparative analysis of the proposed FedPAC-ME framework against standard federated learning baselines, including FedAvg with U-Net, FedProx with U-Net, and an ablation variant using only Multi-Perspective Attention (MPA). The results indicate that FedPAC-ME consistently achieves improved performance across all evaluated metrics when compared to standard FL baselines. In particular, the integration of multi-perspective attention, expert-guided specialization, and perspective-aware contrastive learning contributes to more stable and discriminative representations under federated settings. Specifically, it achieves the highest accuracy of 99.20%, along with an F1-score and Dice coefficient of 99.31%, indicating precise and consistent segmentation and classification performance. Moreover, the model records a confidence score of 96.34%, suggesting that the embedding space learned by the contrastive mechanism is both discriminative and robust. The lowest loss value of 0.0729 further validates the model's efficiency in learning meaningful patterns while minimizing prediction errors. In contrast, FedAvg and FedProx, though effective, show significantly lower performance across all metrics, highlighting the importance of integrating multi-perspective contrastive learning and expert-guided attention. The ablation study with only MPA shows moderate improvements over FedAvg, but still falls short of the full FedPAC-ME pipeline, underlining the complementary contributions of MoE and PACL in enhancing the overall system performance.

All baseline models were trained using the same backbone architecture (U-Net), identical client partitioning, data splits, optimization parameters, and training rounds. This ensures a fair and controlled comparison, isolating the impact of the proposed multi-perspective attention, mixture-of-experts, and contrastive learning components.

Table 8 reports an ablation study designed to quantify the individual contributions of Multi-Perspective Attention (MPA), Mixture of Experts (MoE), and Perspective-Aware Contrastive Learning (PACL) within the FedPAC-ME framework. The full model, FedPAC-ME, achieves the highest performance with an accuracy of 99.20%, Dice coefficient of 99.31, and a confidence score of 96.34, validating the effectiveness of the integrated design. Removing MPA results in a performance drop, with accuracy decreasing to 96.75% and confidence to 91.32%, indicating the importance of attention in capturing critical features from diverse perspectives. Similarly, excluding the MoE component leads to a reduction in accuracy (97.12%) and confidence (92.17%), suggesting that expert specialization helps in adapting to heterogeneous data distributions across clients. The most significant performance degradation is observed when PACL is removed, with accuracy dropping to 95.84% and confidence to 89.56%, highlighting the critical role of contrastive learning in enhancing representation quality and inter-client generalization. These results collectively affirm that all three components, MPA, MoE, and PACL, contribute synergistically to the superior performance of the proposed FedPAC-ME framework.

**Table 8:** Comparison of FedPAC-ME ablation study (accuracy in %).

Model variant	Accuracy (%)	Dice	Confidence
FedPAC-ME (Proposed)	**99.20**	**99.31**	**96.34**
No MPA	96.75	96.88	91.32
No MoE	97.12	97.24	92.17
No PACL	95.84	96.02	89.56

Bold values indicates the highest performance obtained among the ablation variants for each evaluation metric and highlight the results of the proposed FedPAC-ME model.

Overall, these results demonstrate that the proposed FedPAC-ME framework enables robust and reproducible performance improvements in federated medical image analysis while preserving data privacy, making it well-suited for collaborative clinical applications. The privacy-preserving advantages of FL make it a viable solution for collaborative medical research without compromising performance.

## 5 Communication overhead and computational complexity

To comprehensively assess the efficiency of the proposed FedPAC-ME framework, this section analyzes both its communication overhead and computational complexity, comparing model transmission costs and FLOPs against standard federated learning baselines. Together, the communication cost and FLOPs analyses provide a holistic view of the overall resource requirements of FedPAC-ME. While the communication table quantifies the overhead associated with exchanging model parameters during federated training, the FLOPs table captures the computational burden incurred during forward and backward passes.

To provide a clear understanding of the communication efficiency of the proposed FEDPAC-ME framework, Table 9 presents a quantitative comparison of the communication cost across different federated learning baselines. The number of transmitted parameters accounts for both upload and download operations per communication round, while the model size is calculated assuming 32-bit floating-point precision. FedAvg and FedProx, which use the standard U-Net backbone, require the exchange of 34.5 × 10^6^ parameters per round. Incorporating the multi-perspective attention module slighlty increase the communication load to 37.1 × 10^6^ parameters. In contrast, FedPAC-ME introduces additional components, MHSA, MoE, and PACL which increase the total parameter count to 39.2 × 10^6^. This results in a transmitted model size of 1.57 × 10^8^ bytes. Although FedPAC-ME introduces a modest increase of only 13.6% in communication overhead compared compared to the baseline U-net models, this cost is justified by the substantial performance gains observed in accuracy and robustness across clients, demonstrating a favorable trade-off between communication burden and model effectiveness.

**Table 9:** Communication cost of federated learning algorithms.

Method	Params communicated	Model size (bytes)	Global rounds	Time per epoch (s)
FedAvg (U-Net)	34.5 × 10^6^·*e*·*C*·2	1.38 × 10^8^	50	12.4
FedProx (U-Net)	34.5 × 10^6^·*e*·*C*·2	1.38 × 10^8^	50	12.6
MPA-only	37.1 × 10^6^·*e*·*C*·2	1.48 × 10^8^	50	13.1
**FedPAC-ME (Ours)**	39.2 × 10^6^·*e*·*C*·2	**1.57 × 10^8^**	**50**	**14.3**

All methods use 10 local epochs (*e* = 10). Model size in bytes is computed as #parameters × 4 (float32). C denotes the number of participating clients. Global rounds were fixed to 50 for fair comparison. Time per epoch is the average local computation time measured on a single client GPU. Bold values indicate the results corresponding to the proposed FedPAC-ME method and are highlighted for comparision with the baseline methods.

To further assess the computational efficiency of the proposed FedPAC-ME framework, Table 10 presents a comparison of the floating-point operations (FLOPs) required by various federated learning models. All baseline methods, including FedAvg, FedProx, FedBN, FedNova, and FedRS, utilize the same U-Net backbone, resulting in an identical computational cost of 3.454 × 10^11^ FLOPs. Incorporating only the multi-perspective attention (MPA) module introduces a modest increase in computation, raising the total FLOPs to 3.654 × 10^11^. In contrast, the complete FedPAC-ME model integrates MHSA, MoE, and PACL, leading to a total of 3.934 × 10^11^ FLOPs. This represents an approximate 13.9% increase over the standard U-Net configuration. Despite the additional operations, FedPAC-ME achieves substantially higher segmentation performance and improved robustness across heterogeneous clients, demonstrating that the moderate computational overhead is a justified trade-off for the gains in accuracy and generalization.

**Table 10:** FLOPs of federated learning models.

Algorithms	FLOPs
**FedPAC-ME (ours)**	3.934 × 10^11^
FedAvg (U-Net)	3.454 × 10^11^
FedProx (U-Net)	3.454 × 10^11^
FedBN	3.454 × 10^11^
FedNova	3.454 × 10^11^
FedRS	3.454 × 10^11^
MPA-only	3.654 × 10^11^

Bold values indicate the total FLOPs (floating-point operations) of the proposed model, reflecting the addtional computational overhead introduced by the integration MPA, MHSA, MoE, and PACL modules.

Overall, the analysis demonstrates that FedPAC-ME maintains a favorable balance between performance and efficiency. Despite introducing additional modules, the increase in communication and computational cost remains modest, while the resulting gains in segmentation accuracy and cross-client robustness are substantial. These results confirm that FedPAC-ME offers an efficient and scalable solution for federated medical image analysis, making it suitable for deployment in resource-constrained clinical environments.

### 5.1 Ablation study on contrastive learning space

To validate the effectiveness of parameter-level contrastive learning, we compare it with feature-level contrastive learning and a baseline without contrastive alignment. The results in Table 11 indicate that parameter-level contrastive learning achieves improved performance, suggesting that update directions capture meaningful task-relevant information in federated settings.

**Table 11:** Ablation study on contrastive learning space (accuracy in %).

Method	Accuracy (%)	Dice
Parameter-level contrastive (proposed)	**99.20**	**99.31**
Feature-level contrastive	96.3	0.94
Without contrastive learning	95.6	0.92

Bold values indicate the highest performance obtained among the compared contrastive learning methods for each evaluation metric and highlight the results of the proposed approach.

## 6 Discussion

The experimental results suggest that FedPAC-ME is effective in addressing several challenges in federated medical image analysis. By integrating contrastive learning, attention mechanisms, and expert specialization, the framework achieves high segmentation accuracy and low loss, indicating competitive performance relative to centralized approaches while preserving data privacy. However, these findings should be interpreted cautiously, as performance gains are evaluated within a controlled experimental setting.

Despite inter-client variability arising from differences in tumor types, imaging modalities, and data distributions, the global model exhibited relatively stable performance. This indicates the potential of FedPAC-ME to operate under non-IID conditions, which are characteristic of real-world healthcare environments. Nevertheless, such robustness is dataset-dependent, and the absence of external validation limits the ability to generalize these findings across institutions with differing acquisition protocols and patient demographics.

A critical limitation observed across existing federated learning approaches, including the proposed framework, is the reliance on benchmark datasets that may not fully capture real-world variability. Datasets such as BraTS2020 are curated and preprocessed, potentially introducing dataset bias and reducing ecological validity. Additionally, the absence of standardized cross-institutional evaluation protocols makes it difficult to assess true generalizability. Overfitting to specific dataset characteristics remains a concern, particularly in deep learning-based models with high representational capacity.

The MPA, MoE, and PACL modules collectively contribute to performance improvements by enhancing feature representation, enabling client-level adaptation, and promoting alignment across heterogeneous data distributions. However, their individual contributions were not isolated through ablation studies, limiting interpretability of the architecture. This reflects a broader challenge in deep learning systems, where increasing architectural complexity often comes at the cost of transparency and reproducibility.

From a methodological perspective, traditional machine learning approaches, while less expressive, offer greater interpretability and require smaller datasets, making them suitable for constrained clinical scenarios. In contrast, deep learning methods, including federated variants, achieve superior performance but are more susceptible to overfitting, require extensive data, and lack transparency. Hybrid approaches that integrate explainable AI (XAI) techniques or combine classical and deep learning methods have emerged as a promising direction to balance performance and interpretability. While these directions are highly relevant, their integration into federated semi-supervised frameworks remains an open area for future research.

Recent work such as Gagliardi et al. (2025) highlights the growing importance of hybrid explainable AI frameworks that combine deep learning with interpretable features for brain MRI analysis. Such approaches improve clinical trust and transparency, which remain critical challenges in federated medical imaging. Hybrid architectures combining convolutional networks with transformers have shown promising results in visual tasks, even beyond medical imaging domains (Gangwar et al., 2024). These models leverage both local feature extraction and global context modeling, suggesting potential extensions for federated medical imaging frameworks. While the proposed FedPAC-ME framework focuses on privacy and representation learning, integrating explainability mechanisms could further enhance its clinical applicability.

Several limitations specific to this study should be acknowledged. First, the evaluation was conducted solely on the BraTS2020 dataset, which focuses on brain tumor segmentation. As such, the applicability of the framework to other imaging modalities and clinical tasks remains unverified. Second, repeated experimental runs and statistical significance testing were not performed due to computational constraints, limiting the robustness of the reported results. Third, the use of a 2D slice-based approach, while computationally efficient, restricts the model's ability to capture full volumetric context, which may impact performance in anatomically complex regions.

From a deployment perspective, federated learning systems face several practical challenges that are not addressed in this study. These include communication overhead, training latency, client availability, and system heterogeneity. Additionally, reproducibility remains a concern due to variability in local training conditions across clients. Domain shift across institutions, arising from differences in imaging devices and protocols, further complicates deployment and may degrade model performance. Regulatory and privacy constraints, particularly in healthcare, also impose limitations on data sharing, model validation, and clinical adoption.

DenseNet-121 was selected as the backbone due to its strong feature extraction capabilities; however, its computational complexity may limit deployment on resource-constrained clients. Although strategies such as image downsampling and sparse expert activation were employed, further optimization is required. Future work will explore lightweight architectures and adaptive model scaling techniques to improve efficiency without significantly compromising performance.

While the proposed framework is designed to be modality-agnostic, this claim remains speculative without empirical validation on diverse datasets. Extending evaluation to modalities such as CT and ultrasound, as well as multi-center datasets, will be essential to establish robustness and generalizability.

Finally, although FedPAC-ME incorporates mechanisms that suggest scalability, including modular expert design and localized contrastive learning, its performance under large-scale federated settings with many clients remains untested. Future work will focus on evaluating scalability, communication efficiency, and system-level performance under realistic deployment conditions.

## 7 Conclusion

This work presents FedPAC-ME, a federated learning framework for privacy-preserving medical image classification that integrates Multi-Head Self-Attention (MHSA), Mixture of Experts (MoE), and Perspective-Aware Contrastive Loss (PACL). The proposed framework is designed to enhance feature representation, adapt to heterogeneous data distributions, and improve alignment across decentralized clients.

Experimental results on the BraTS2020 dataset demonstrate that FedPAC-ME achieves competitive performance across multiple evaluation metrics, including Dice score and F1-score, under non-IID conditions. The model shows the potential to approach the performance of centralized methods while preserving data privacy, highlighting the effectiveness of combining attention mechanisms, expert-based personalization, and contrastive alignment in federated settings.

Despite these promising results, several limitations must be acknowledged. The evaluation is restricted to a single dataset and specific experimental configurations, and statistical significance analysis was not conducted. Therefore, the observed performance may not generalize to other datasets, imaging modalities, or clinical tasks. Additionally, variations in client data distributions and training conditions may affect model performance in real-world deployments.

Future work will focus on expanding evaluations to more diverse medical imaging datasets, such as chest X-rays, dermatological images, and cardiac MRI, along with incorporating statistical validation through repeated experiments. Further investigation into system-level aspects, including communication efficiency and scalability, will also be important for practical implementation.

Overall, FedPAC-ME provides a promising step toward privacy-preserving federated medical image analysis. However, broader empirical validation and system-level evaluation are required to fully assess its robustness and applicability in clinical settings.

## Data Availability

The original contributions presented in the study are included in the article/supplementary material, further inquiries can be directed to the corresponding author.
